# A novel method to assess spatio-temporal habitat availability for a generalist indicator species group in human-modified landscapes

**DOI:** 10.1007/s10980-025-02124-x

**Published:** 2025-05-17

**Authors:** Nivedita Varma Harisena, Adrienne Grêt-Regamey, Maarten J. van Strien

**Affiliations:** Planning of Landscape and Urban Systems PLUS, Department of Civil, Environmental and Geomatic Engineering, ETH Zurich, Stefano-Franscini-Platz 5, CH-8093 Zurich, Switzerland

**Keywords:** Habitat availability, Spatio-temporal, Landscape history, Indicator species, Human-modified landscape

## Abstract

**Context:**

Landscape changes can alter habitat availability for species over time. There can be a time-lagged response of species to such changes, leading to possible extinction debts. In human-modified landscapes, understanding these dynamics is critical to inform conservation actions and mitigate biodiversity loss.

**Objectives:**

This study examines temporal trajectories of habitat availability over 113 years from 1899 to 2012 in the Swiss Plateau and evaluates their relationship with current occurrences of an indicator generalist species group that inhabits mosaic agricultural landscapes.

**Methods:**

Time-series of resistance surfaces were derived from roads and buildings. Resistance kernels were then used to calculate the Amount of Habitat Available (AHA) metric across five maximum dispersal distances. Spatio-temporal patterns of AHA were analysed using multi-dimensional K-Means time-series clustering. The clusters were evaluated based on their overlap with species occurrences. The suitability of AHA to predict species presences was also determined. The results were compared with current best-practice approaches that use contemporary landscape data and fixed-shape moving-windows.

**Results:**

Ten AHA trajectories were identified, showing variable patterns of decline in AHA over time. Time-series clusters with higher historical AHA were associated significantly with greater contemporary species occurrences. The AHA in 1933 showed the strongest link to current species presences, highlighting a time-lagged response. The presented approach outperformed the current best-practice approaches.

**Conclusions:**

Historical trajectories of habitat availability are essential for understanding species occurrences and time-lagged responses to landscape changes. The presented approach is generic and effectively links historical dynamics to current biodiversity, supporting conservation planning in human-modified landscapes.

**Supplementary Information:**

The online version contains supplementary material available at 10.1007/s10980-025-02124-x.

## Introduction

Biodiversity depends on a complex interplay of habitat area and connectivity placed within the broader background of landscape history (Cristofoli et al. 2010; Ewers et al. 2013; Herrault et al. 2016). Both habitat loss and fragmentation are not static processes that are captured in a single moment in time but are part of a longer trajectory of landscape change (Rogan et al. 2023). Tracing such trajectories of landscape change over time, especially within human-modified landscapes, can provide critical information on time-lagged responses of species to such change (Tappeiner et al. 2020; Liao et al. 2022).

Historical landscape transformations impact current species occurrence through time-lagged processes, like extinction debt or via a path dependence on historical landscape structure (Essl et al. 2015b; Tappeiner et al. 2020; Pan et al. 2022; Harisena et al. 2024). Shrinking habitats may show a surplus of species relative to what is expected based on the species-area relationship, indicating delayed extinctions to be "paid off" in the future (Guardiola et al. 2013). Additionally, when species-area relationships do not reach equilibrium, as the landscape increases in human modification, then species continue to persist based on their sensitivity to human modifications (Martins & Pereira 2017). Such extinction debts have been traced from decades to more than a century into the past (Scherreiks et al. 2022; Harisena et al. 2024; Zhang et al. 2024). Areas in this transient state of delayed extinction require conservation initiatives at par with historical landscape conditions to allow for a pause or reversal in species loss (Essl et al. 2015a). It is thus important to identify trajectories of landscape change when analysing contemporary species occurrences.

A major underlying mechanism that defines how a landscape impacts species diversity is by the amount of habitat available for a species at a certain location (Saura & Rubio 2010; Fahrig 2013). To quantify ‘habitat availability’ one must determine how much habitat is reachable by individuals from a certain species, which is affected both by the dispersal ability of a species as well as the landscape conditions that hinder or facilitate dispersal (Saura & Pascual-Hortal 2007; Reider et al. 2018). A landscape heavily modified by factors of increasing human development and activity, including built-up land use, traffic, noise and light, can affect animal movement (Rivki et al. 2007; Northrup et al. 2016). As the human modification of the landscape increases over time, the ability of a species to reach habitats of its preference is reduced (Beyer et al. 2010). Contrastingly, there can also exist a time-delayed response of species in such changing landscapes dependent on their sensitivity to human modifications, leading to continued persistence in modified landscapes (Martins & Pereira 2017). In summary, habitat availability in the local surroundings for a species can be considered a function of 1. dispersal capacities of the species, 2. local conditions in its vicinity; and 3. changes in the landscape over time.

Most studies that quantify the habitat available to a species use a patch-based approach. In such studies, discrete habitat patches are identified in the landscape and habitat availability for a species is calculated based on the network distance between such predefined patches (Saura & Pascual-Hortal 2007; Saura & Rubio 2010; Diniz et al. 2018; Khiali-Miab et al. 2022). For example, the ‘probability of connectivity’ calculates the habitat available as a product of individual patch areas weighted by the probability of dispersal between the patches (Saura & Pascual-Hortal 2007). However, many species, especially generalists, do not have a clear preference for a certain habitat type and inhabit a mosaic of different types of habitats (Quinn et al. 2013; Macchi et al. 2020). For these species, estimating habitat availability is challenging with conventional patch-based approaches, as they require the delineation of discrete habitats in a landscape (Martins & Pereira 2017; Paise et al. 2020). Further, as many patch-based approaches are developed with one or few species in mind, they provide limited understanding of habitat availability for larger groups of species, especially those beyond specialist or umbrella species (Cushman & Landguth 2012). For generalist species groups, it is important to develop alternative methods for estimating habitat availability that are not tied to specific habitat types. Such approaches are particularly useful in mosaic landscapes where human modification varies along a gradient over time (Theobald 2010; Belote et al. 2022). In this context, we focus on Guild 25—a group of generalist indicator species inhabiting heterogeneous agricultural landscapes within the Swiss Plateau (Rutishauser et al. 2023). Their reliance on complex landscape mosaics makes them well-suited for gradient-based assessments of habitat availability beyond traditional patch-based definitions. We use the term “indicator species” to refer to this guild.

One way to calculate habitat availability as a response to human modification is via the use of resistance kernels calculated from landscape resistance surfaces (Compton et al. 2007; Kaszta et al. 2019). Landscape resistance surfaces capture the magnitude to which different land covers impede or facilitate dispersal and therefore can be used to estimate how far an individual organism can move through the landscape. A generic way to parameterise resistance surfaces is to relate the resistance values to the degree of human modification of the landscape, whereby natural or undisturbed areas get a low resistance and built (infra)structure gets high resistance (Zeller et al. 2024). Resistance surfaces are commonly used to estimate the habitat connectivity between habitat patches (Zeller et al. 2012; Cushman et al. 2013a, b) or mimicking movement through pixels likened to current flow (Belote et al. 2022), but may also prove useful to estimate how much area is available for a species that does not have a specific habitat preference. Least cost dispersal kernels calculated on resistance surfaces (a.k.a. resistance kernels) estimate the omni-directional extent of possible dispersal for an organism that starts from the centre of the kernel. Most studies that use these resistance kernels often focus only on connectivity impacts and do not directly provide amount of habitat available for species in its vicinity (Cushman et al. 2013a, b; Diniz et al. 2020). However, such kernels can also be a useful tool to estimate the amount of habitat available in the local surrounding of a species (Eigenbrod et al. 2008; Fahrig 2013). Additionally, a range of cost-distance thresholds, signifying the varied dispersal abilities in a species group, can be included to define the resistance kernels. Unlike fixed shape moving windows, such kernels change shape and size based on landscape resistance. Mapping such kernels for each cell in a landscape raster creates a geographically continuous estimation of available habitat for species groups, bypassing strict habitat definitions.

To summarise, this study has two primary aims. First, it seeks to understand how temporal trajectories of habitat availability influence the occurrence of generalist species in the Swiss Plateau. For this we investigate a group of indicator species that prefers mosaic landscapes of richly structured meadows and cultivated patches, as defined by Rutishauser et al. (2023), within the Swiss Plateau. Second, to conduct this analysis, we introduce a novel metric—the *Amount of Habitat Available* (AHA)—which quantifies available habitat based on species’ dispersal capacity within a resistant landscape. We compute AHA for both contemporary (2012) and historical (1899–1992) periods using land cover data digitized from historical maps. We hypothesize that areas with greater historical habitat availability currently support higher species occurrences. Finally, we discuss the broader applicability of this approach for modelling habitat availability within species’ dispersal constraints as compared to current best practices and emphasize the importance of incorporating historical context into such analyses.

## Methods

### Study area

This study was conducted within the biogeographical region of the Swiss Plateau (BAFU, 2022), excluding peripheral areas near Basel and Geneva to minimize edge effects and due to incomplete historical data. Given the region’s topographic homogeneity, it is a reasonable assumption that species dispersal is primarily influenced by human-made barriers. Therefore, to parameterise our resistance surfaces, we included spatio-temporal information on roads and buildings, as these are the most prominent human-made barriers in the Swiss Plateau.

### Road and building information for the different time-steps

The first step in the analysis involved the cleaning and aggregation of the building and road maps for the study area for the eight time-steps of 1899, 1918, 1933, 1959, 1970, 1978, 1992 and 2012. The building and road information for the historical time-steps were segmented from cartographic maps: for 1899–1949 we use information from Siegfried maps (Swisstopo 2024b); for 1959–1992 we use information from the Old National maps (Swisstopo 2024a); and for 2012 time-step we use the Swiss TLM map published by Swisstopo (Swisstopo 2023). The methodology for the choice of these eight time-steps, the map sheets chosen and the segmentation of roads and buildings is described in Räth et al. (2023).

Building data, provided in vector format, were converted to rasters for analysis. Roads were segmented into raster outputs at 1.25 m resolution, representing the probability of road presence. A visually validated threshold was applied to generate binary presence-absence layers, minimizing noise from cartographic artifacts (see Supplementary S1). The road segmentation from both the Siegfried and Old National maps were comparable and the same data cleaning method could be repeated. An important characteristic of this cartographic data includes the use of different line thicknesses for denoting different road types, varying from 10 to 50 m with higher (and usually busier) road types being widest. These values are in line with the typical road widths for different road types for motorised traffic (Swisstopo 2023).

Along with buildings and roads, lakes were also included in the landcover information. Since significant changes in lake extents were not expected over the 113-year period prior to 2012, the spatial extent of lakes from the most recent dataset was applied uniformly across all time steps. These data cleaning and aggregation for the entire Swiss plateau and across 7 time steps (1899–1992) was done using the R package Terra (Hijmans 2024; R Core Team 2024).

For 2012, vector road and building data were rasterized to ensure consistency with historical layers. Roads from this data were buffered based on certain widths identified from historical equivalents through visual inspection (see Supplementary Table S2).

### Indicator species data

We investigated whether spatio-temporal trajectories of habitat availability can explain the occurrence of indicator species. To select our indicator species group, we used one of the guilds defined by Rutishauser et al. (2023), who grouped species in Switzerland with similar habitat requirements based on expert knowledge. These guilds reflect habitat quality through the presence of indicator species. We focused on Guild 25—a distinct group of mobile vertebrate species that inhabit extensive, structurally rich cultural landscapes such as meadows, arable land, and perennial crops. The guild includes 52 species of birds, amphibians, reptiles, and small mammals (see Supplementary Table S3). All are mobile and are either threatened, characteristic of these habitats, or serve as indicators of their biodiversity potential. From an ecological perspective, they exhibit more complex habitat requirements than sessile organisms and rely on a mosaic of interconnected habitats distributed across the landscape. Their generalist nature and reliance on spatially heterogeneous landscapes make them suitable for evaluating continuous patterns of habitat availability.

A nationwide raster map (100 × 100 m resolution) indicating landscape quality for this guild was provided by Rutishauser et al. (2023; see Supplementary Material S3). Based on non-systematic observations since 2000, the map reflects species presence within a 3 × 3 pixel neighbourhood, accounting for their mobility. A quality value of ‘1’ indicates presence in one cell, while a value of ‘9’ indicates presence in all nine. In our analysis, values of 5 or above represent ‘high quality areas’—pixels with multiple nearby observations—while any value of 1 or higher is treated as presence data. This presence metric does not reflect species richness but indicates that at least one species of the indicator list occurs, which experts regard as sufficient to infer habitat quality (Rutishauser et al. 2023).

Since the sizes and dispersal abilities of the species in the guild vary, a multi-scale approach is necessary when estimating habitat availability. Therefore, we tested dispersal distance thresholds of 250, 500, 1000, 2000, and 4000 m. These distances were chosen to reflect the range of movement capacities, in a landscape without resistance, for the indicator species group, which contains both species with a body mass of several kg as well as smaller-bodied species, such as reptiles and amphibians. In a comprehensive review of species dispersal distances, Jenkins et al. (2007) found a relationship between body mass and dispersal distance. The chosen values reflect the range of dispersal distances plausible for our chosen indicator species. Even though species with similar body sizes in our group have been recorded to move up to 10 km (Jenkins et al. 2007), we did not include any distances beyond 4000 m, as preliminary analysis showed negligible variation in habitat availability estimates at larger thresholds.

### Calculating landscape resistance

From the historical land cover data, we developed landscape resistance surfaces at 10 m resolution in which we assigned resistance-to-movement values to roads and buildings. The resistance values are indicative of the amount of effort taken to circumvent or cross barriers in the landscape by the species. We utilised a methodology as defined in Shirk et al. (2010) to quantify the landscape resistance values:

$$C_{i} = \left( {\frac{{Rank_{i} }}{{Rank_{{max}} }}} \right)^{x} \times C_{{max}}$$

where ${C}_{i}$ is the cost of the* i*^*th*^ landcover class. ${Rank}_{i}$ is the rank of a landcover, in terms of barrier to movement, compared to the highest barricading landcover ${Rank}_{max}$, which in our case is 3. ${C}_{max}$ is the maximum cost assigned to this ${Rank}_{max}$ landcover. *x* takes a value within [1,2,4,8 or 16] each defining the magnitude of the contrast between ${Rank}_{i}$ and ${Rank}_{max}$ landcovers (Savary et al. 2021). We chose an average contrast scenario ($x$ = 4) where the second ranked landcover receives almost half the cost as the highest (highlighted in Supplementary table S4).

For the ranking of the landcover we use the following logic: being mobile and generalist, we assume that the indicator species experience minimal resistance across any open or natural areas. Hence, we did not differentiate resistance values between forests, open land and wetlands and these are given the lowest ranks (i.e. no barrier). Roads were considered semi-permeable, i.e. they can be crossed, however, they can reduce dispersal due to mortality or road avoidance due to noise or traffic and are given a rank of 2.5 to indicate slightly less cost than buildings (given rank 3). For each road pixel, a fixed resistance value was given, however, as the width of the roads varied according to road type, wider – and usually busier – roads accumulatively have a higher resistance than narrower roads. As detailed in Supplementary Material S1, the historical road data distinctly illustrates varying road widths. Buildings were assumed the highest rank as barriers since the effort to circumvent around buildings is higher. Lakes were assumed to be barriers with a low rank of 2 since certain amphibian species were included and most habitat could lie alongside lakes.

**Fig. 1:**
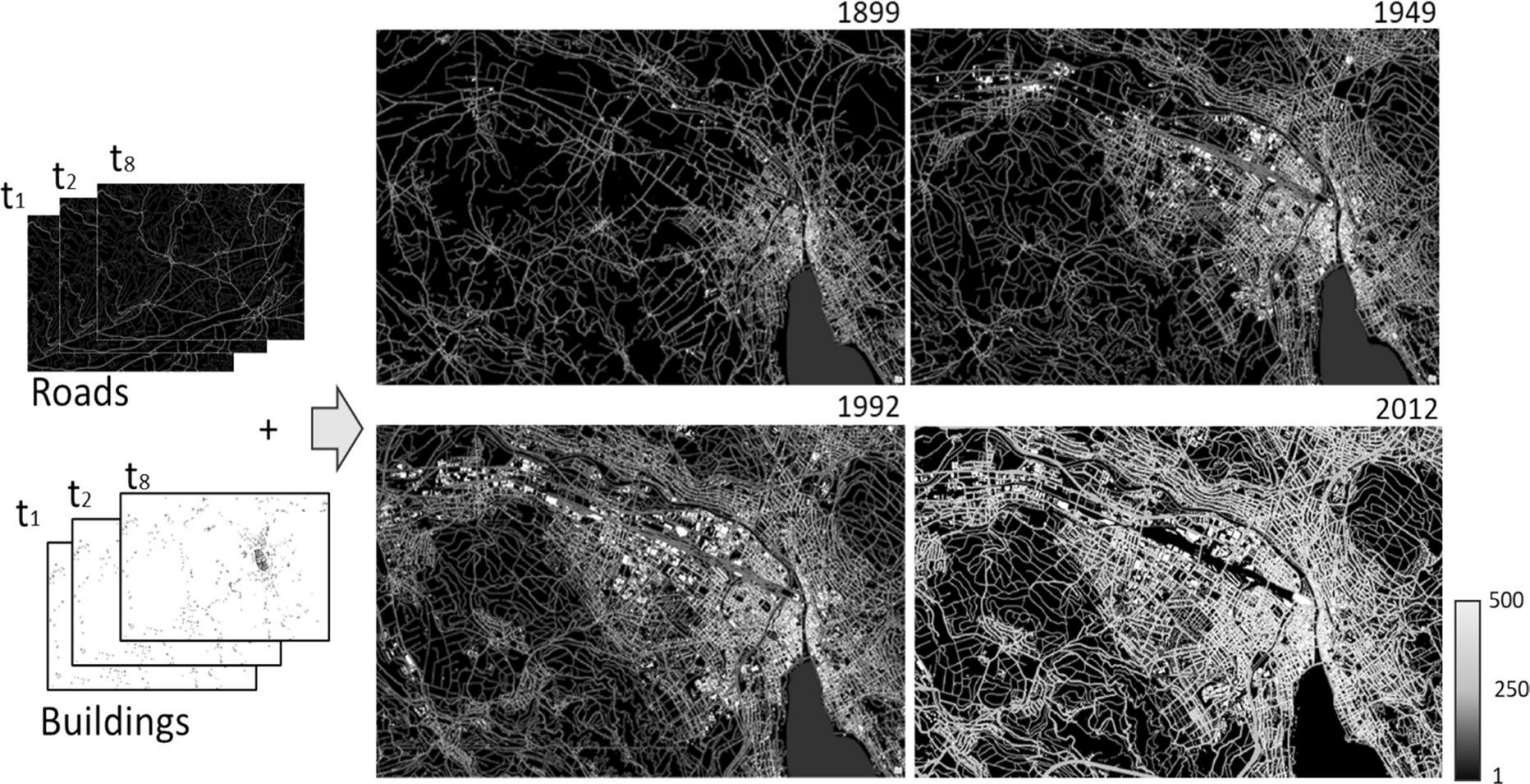
Resistance surfaces created from historical time-series of buildings and roads for an example area around the city of Zurich. Resistance surfaces were created for eight time-steps (t1, t2, …., t8). Buildings have highest resistance of 500 whereas roads have a value of 250 and the rest of the areas are fully permeable

Given the above-mentioned rankings, average contrastive scenario and a highest cost of 50 per 10 m pixel (therefore 500 units per pixel), the relative per pixel resistance values were assigned for the landcover data (See highlight in Supplementary table S4). These resistances were calculated for each time-step as mentioned previously. Figure 1 presents the resistance surface calculations for a sample area near Zurich at four selected time steps.

### Calculating Amount of Habitat Available (AHA)

**Fig. 2:**
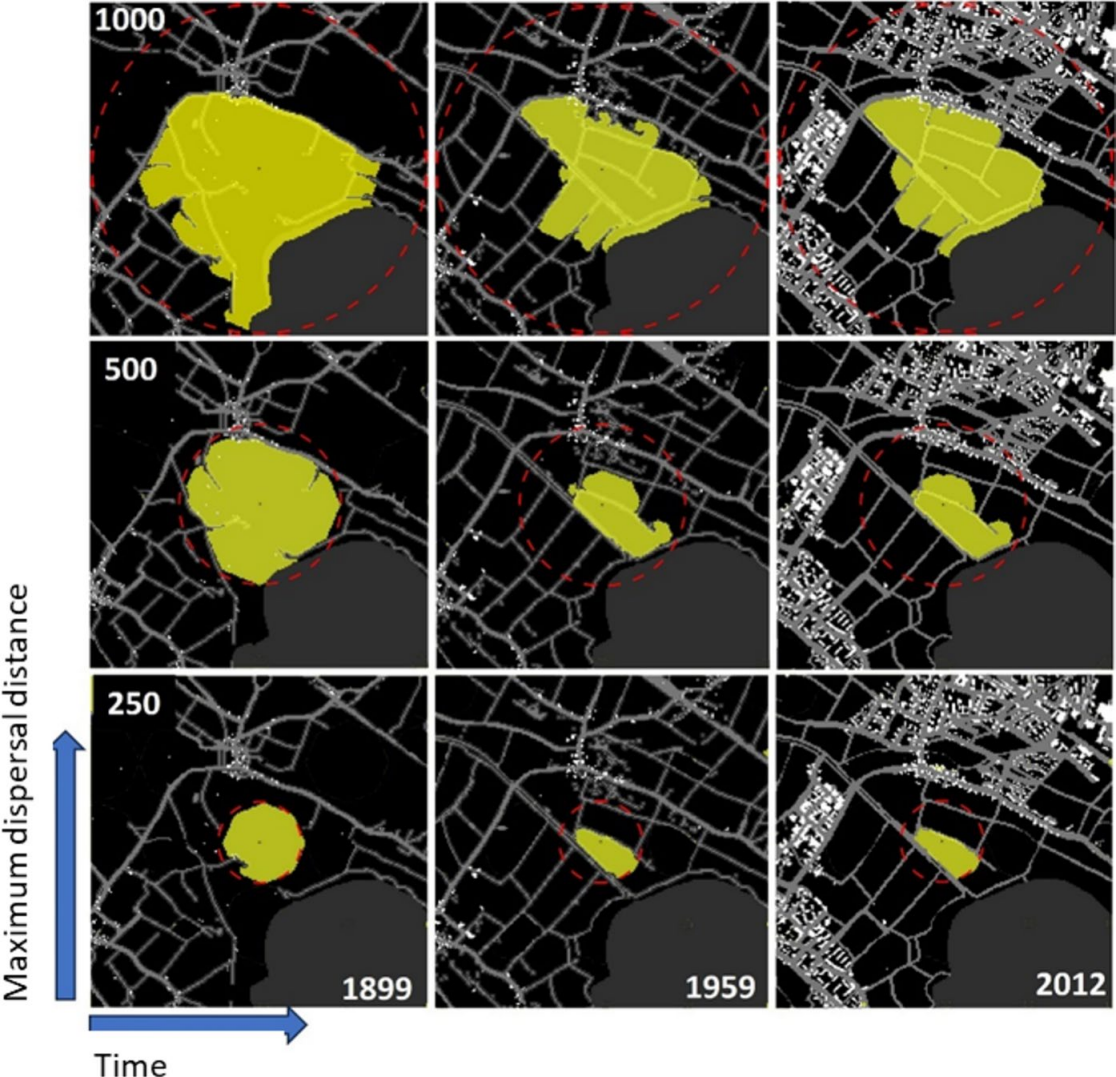
Examples of resistance kernels around a focal point within an exemplary resistant surface in the Swiss plateau. The yellow is the resistance kernel truncated for different maximum dispersal distances (y-axis) and different points in time (x-axis). Buildings and roads provide resistance to dispersal (shown in white). The red dotted line indicates the hypothetical maximum movement area given homogenous favourable landscape without resistance. The calculations were repeated for five different dispersal distances (y-axis) and eight time-steps (x-axis)

The Amount of Habitat Available (AHA) metric is a continuous measure of habitat availability across the landscape. For computational reasons, and because of our lowest dispersal distance choice, the cost distance kernels were calculated for each centre point in a regular grid of 250 m resolution across our case study area. For each 250 × 250 m grid, AHA is calculated using cost distance kernels applied to resistance surfaces at 10 m resolution, estimating how much surrounding habitat is reachable from the pixel’s center. Cost values accumulate radially from the center based on landscape features, with movement truncated at a maximum cost reflecting species’ dispersal ability (250, 500, 1000, 2000, and 4000 units). For example, a species with a dispersal capacity of 500 units could traverse 500 pixels of meadows, or fewer if encountering higher-cost barriers like roads or buildings. Pixels within this cost limit are classified as available habitat. By computing AHA for multiple dispersal thresholds, we assess habitat availability at different ecological scales. These estimates are sensitive to both the density and configuration of landscape barriers, as shown in Fig. 2, and allow us to evaluate how landscape structure influences habitat accessibility over time.

The available habitat extent was then converted to a polygon, its area calculated, and the resulting value assigned to the corresponding centre-point pixel. We propose the metric the Amount of Available Habitat ${AHA}_{p}$ where $p$ is a 250 by 250 m pixel in the landscape based on the following formulation:

$${AHA}_{p}=\frac{{a}_{p}}{{\pi R}^{2}}$$

Here ${a}_{p}$ is the area of the extent up to which the species can disperse in a resistive landscape (yellow extent in Fig. 2), $R$ is the radius of the hypothetical maximum area a species can cover in a homogeneous non-resistant landscape (red dashed circle in Fig. 2). This ratio makes the habitat availability comparable across dispersal distances. We calculate $AHA$ for each *p*^*th*^ pixel across eight time-steps (1899, 1918, 1933, 1959, 1970, 1978, 1992, and 2012) for the entire study area (Fig. 2). For each pixel and each time-step, there are thus five AHA values: one for each maximum dispersal ability. A detailed workflow of this AHA calculation method is shown in Supplementary material S15.

### Time-series clustering

We use the AHAs calculated per 250 m-by-250 m grid for each time step and each maximum dispersal distance as input for time-series clustering. We identify pixels with similar trajectories of change in the calculated AHA across the five dispersal distances and eight time-steps using a KMeans based multi-dimensional time-series clustering approach as implemented in the tslearn package in python (Tavenard et al. 2020). This is an unsupervised clustering algorithm that groups data points by minimising the Euclidean distance between different time-series. The KMeans algorithm first randomly initialises centroids for the dataset, and based on multiple iterations it assigns each time-series data point to the nearest centroid till the overall variance within the clusters in terms of Euclidean distance to cluster centroid is minimised. This is then repeated for multiple random initial centroids. This minimisation occurs across all dimensions and time points. Therefore, data points in a cluster have similar temporal change in AHA profiles across all dispersal distances.

One of the important parameters in such clustering methods is the definition of the optimum number of clusters. The optimum number of clusters is identified by plotting and comparing the change in Within-Cluster Sum of Squares (WCSS) as the number of clusters increases (Hartigan & Wong 1979). The WCSS averages the total distance between each time-series data point and its corresponding cluster centroid i.e. the within cluster dispersion. The lower the WCSS value the more compact the clusters are. The elbow method is used to identify the optimal number of clusters and represents the point in which adding more clusters creates minimal change to the WCSS. Using these methods, we calculated the clusters for each pixel and map them spatially to show clusters of trajectories of change in the study area.

In addition to the time-series cluster, we also performed a clustering on the AHA values of the latest time step (2012), to analyse whether the temporal data adds definitive information to the time-series clustering. We applied a KMeans spatial clustering on AHA values only from the year 2012 across the five dispersal thresholds. This is similar to the time-series clustering, only that a single time-step is used for the clustering. We also created a map of these clusters and henceforth refer to it as “Spatial clustering”.

To improve the comparability of the two clustering approaches, we find the best correspondence between the spatial and the time-series cluster labels. For this we use the Kuhn-Munkres algorithm (Munkres 1957), which minimises a ‘cost matrix’ of the misalignments or number of non-overlapping cluster definitions between the two clustering outputs. A perfect alignment between the two clusters is not likely and therefore we also calculated a confusion matrix between the labels from the two clustering methods.

To examine whether there are relationships between habitat availability trajectories and changes in the human modification density of the landscape, we also calculated Human Modification Densities (HMD) for each pixel. The HMD is defined by the percentage of pixels that are road or building within moving windows of multiple sizes, where the size of each window corresponds to the chosen maximum dispersal distances. HMD was calculated using PyTorch (Paszke et al. 2019).

### Relating to species data

To validate the identified time-series clusters, we assessed whether landscape quality areas—based on the presence of at least one indicator species—occurred more frequently within specific clusters. We compared this with the spatial clustering output. Boxplots were used to visualize the distribution of landscape quality values within each cluster for both clustering methods. Additionally, we calculated the percentage area of high-quality pixels (values 5–9) within each cluster, relative to their total area across the Swiss Plateau, and presented the results as barplots. These barplots highlight how high-quality habitats are distributed among the clusters.

To determine whether clusters significantly overlapped with species presence, we performed a permutation test. We randomly shuffled cluster labels 1000 times, preserving the number of pixels per cluster, and recorded species presence counts in each. Presence was defined as any landscape quality value of 1 or above). Since the pixels of landscape quality indicate the presence of at the least on indicator species, using this data is valid as a presence record for our chosen species group. However, it is a relevant caveat that not all the presence points indicate each of the species in the indicator list. A cluster was considered significant if the observed number of presences was greater than in 99% of the permutations (significantly overlaps), or less than in 99% (significantly deters).

We also investigated how the AHA values per centre point directly related to species presences and absences (i.e. not considering the time-series clusters). Using Generalised Linear Models (GLM’s) with binomial distribution, we explained the landscape quality data converted to presence-absence with individual AHA values for each year and each scale in independent models. We additionally included a combined model (‘Allscales’) for each time-step that includes all the five dispersal-thresholds per year as explanatory variables. We determined the ROC-AUC of all the models across the different years to identify how AHA of multiple spatial scales distinguishes the species presence-absence data. We also calculated the effect size of the predictor in the GLM indicating the increase in probability of occurrence of at least one indicator species per standard deviation change in predictor. To contrast with moving window analyses which is frequently used in such density analyses, we computed the same for models of species presence-absence using the HMD values per centre point. To reduce spatial autocorrelation, the data was subsampled to 30% the original. Given the low species presence rate (20%), the subsample was stratified to include 50% presence and 50% absence cases to ensure balanced model inputs. This sampling approach was repeated 25 times, and results are reported as mean, minimum, and maximum across iterations. GLMs were also fitted to the full dataset, with results included in the Supplementary material.

The modelling of the species data along with the historical landcover cleaning and permutation tests were done in R (R Core Team 2024), whereas the AHA calculations, HMD calculations and the time-series clustering was done in Python (Python Software Foundation 2020). Details of this along with the workflow used to calculate AHA are given in Supplementary section S15. We have also made the code for the AHA calculations available online (See data availability).

## Results

### Time-series clustering

**Fig. 3:**
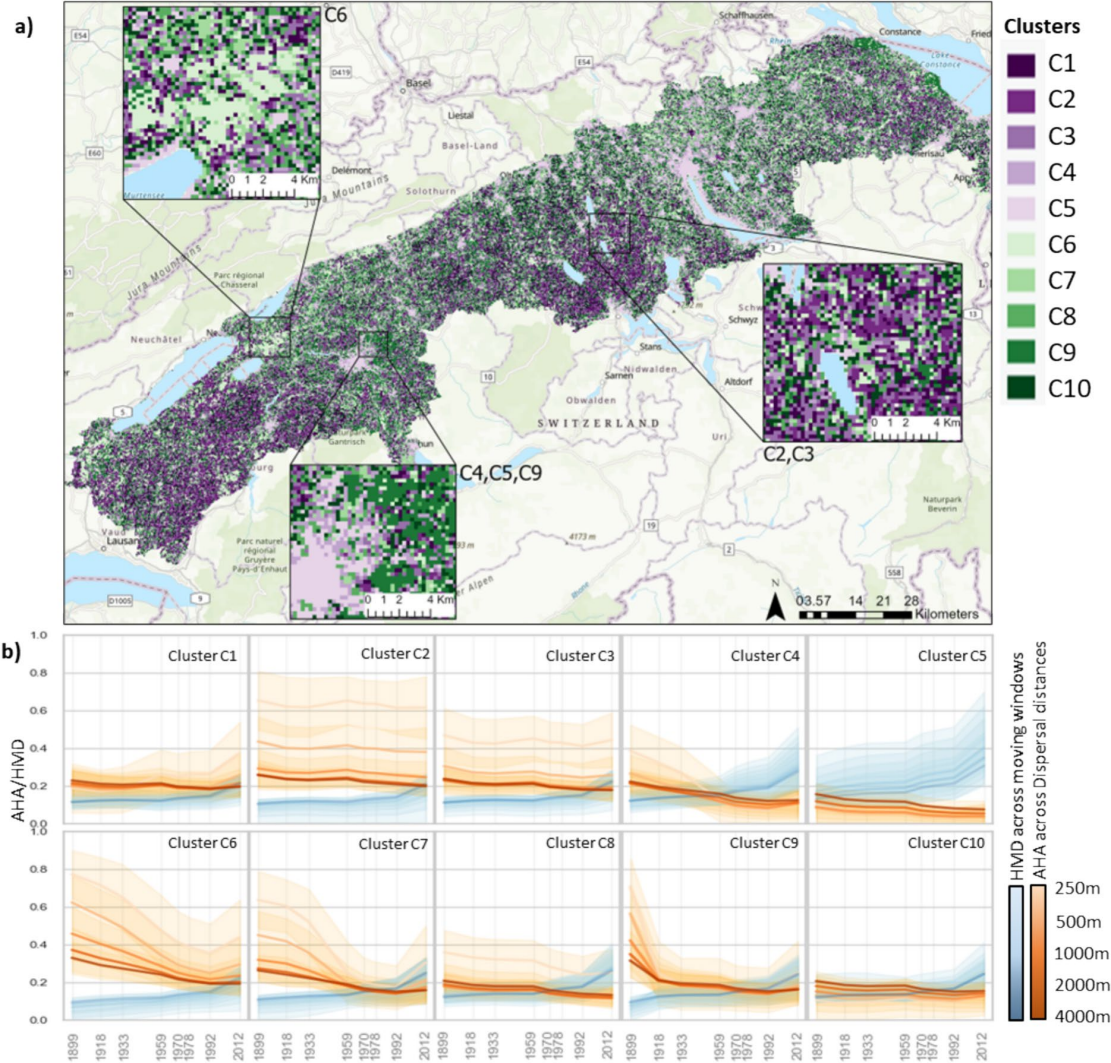
**a** Map of the time-series clusters; **b** The resulting mean values for both the Available Habitat Area (AHA; orange) and Human Modification Density (HMD; blue) for the ten time-series clusters across the five dispersal distances. The shaded area around each line shows the standard deviation of the values across all the pixels that fall within each cluster for which the mean is the solid line. Insets in (a) show zoomed in regions showing significant visual patterns in clustering. The background topographic map is from Esri, TomTom, Garmin, FAO, NOAA. (2017)

The optimum number of clusters identified for the time-series and spatial clustering of the AHA values are 10 and 8, respectively (See Supplementary material S5). The time-series clustering identified ten clusters of trajectories of AHA change over the past 113 years in the Swiss plateau before 2012 (Fig. 3a). The clusters contain distinct trends of AHA over time and over the different maximum dispersal distances (Fig. 3b). In all clusters, we can see that AHA is decreasing over time for all maximum dispersal distances. Across dispersal distances, smaller distances (250, 500 m) show greater variability, as expected in a densely populated region where long-distance dispersal is constrained. Clusters C2 and C3 show only slight decreases, with C2 maintaining consistently higher values. In contrast, clusters C4, C6, C7, and C9 exhibit sharp declines, primarily post-1933 (except cluster C9). Notably, cluster C6 has the highest AHA values for each dispersal distance in the earliest time-step of 1899 (see Supplementary material S5). Custers C6 and C7 additionally show high AHA in the initial time-steps before 1933, followed by a sharp decline after this time step, with cluster C7 declining faster than C6. Cluster C5 shows the lowest values throughout, denoting areas of already saturated settlement densities where additional increases in roads or buildings only slightly declines AHA further.

When comparing the HMD to the AHA trajectories, the trends of AHA are not consistent with the trends in HMD. In cluster C6 and C7 the sudden decrease in AHA at a certain time step cannot be correlated to a similar pattern in the development of HMD. Similarly in cluster C2, C3 there is recent increase in HMD, yet the trend in AHA values remain consistent. Clusters C5 shows relatively low and stable AHA values despite strongly increasing HMD.

The spatial map of the time-series clusters is shown in Fig. 3a. Clear patterns emerge from a visual inspection. Firstly, cluster C5 clearly depicts settlement extents, showing a consistently low AHA over time (Fig. 3b). This cluster is seen to be spatially surrounded by cluster C9 and cluster C4, which show a decrease in AHA post 1899 and 1933, respectively, showing some form of suburban development in the early twentieth century in the Swiss plateau (Fig. 3a; inset C4,C5,C9). Cluster C6 is seen in two small clumps across the Swiss plateau (Fig. 3a; inset C6), depicting a higher AHA in the past (Fig. 3b). Cluster C2,C3 showing areas of quite stable AHA over time (Fig. 3b) are found in the 3 distinct locations (south-west, central and north-east) in the Swiss plateau, one of which is shown in Fig. 3a; inset C2,C3.

No noticeable spatial patterns can be visually detected for the rest of the clusters. The map of spatial clustering shows fewer distinct patterns visually, with only cluster C5 (i.e. urban areas) seen distinctly (See Supplementary material S7).

### Relating clusters to landscape quality

**Fig. 4:**
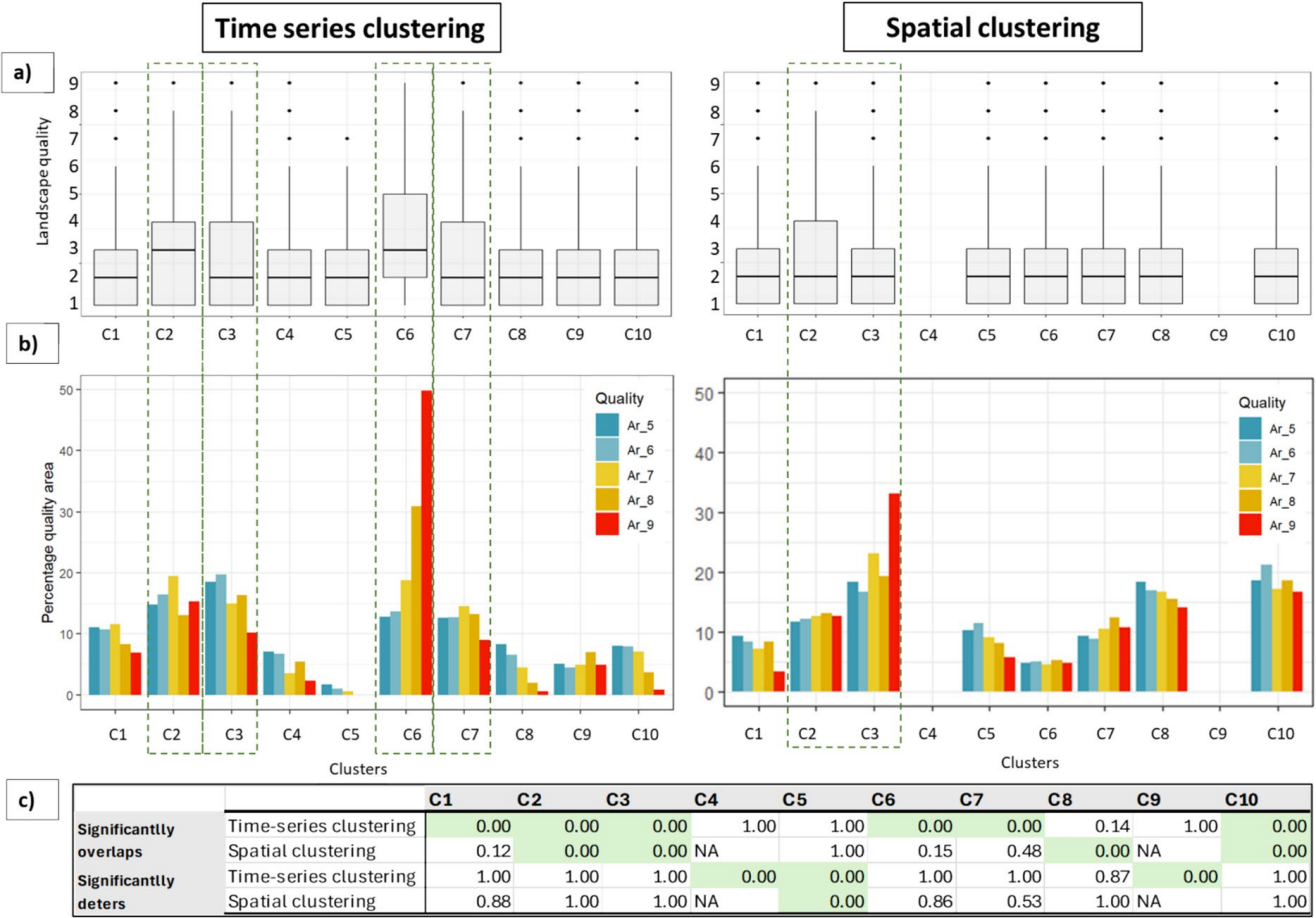
**a** Boxplots of distribution of landscape quality values based on species occurrences for the different clusters; **b** Percentage quality area within the total cluster area in the Swiss plateau for quality categories of five and above. The dotted lines indicate clusters showing higher percentage landscape quality area compared to the other clusters; **c** The significance (99% CI) of the clustering based on how much it significantly overlaps and how much it significantly deters species when compared to a random shuffling of the cluster labels 1000 times. Green denotes significant clusters

Figure 4 shows the distribution of the landscape quality values across the different clusters both from the time-series and spatial clustering methods. The cross-tabulation of the two cluster labels to gain the highest probable overlaps resulted in clusters C4 and C9 from the time series clustering not occurring in the spatial clustering (See Supplementary material S9 for the confusion matrix). Figure 4a and Fig. 4c include data from all landscape quality levels whereas Fig. 4b only shows qualities above 4 since the distinction in the distribution of areas is evident in these higher quality areas.

The distribution of landscape quality values of the chosen indicator species in clusters C2 and C6 have a higher median than in the rest of the clusters (Fig. 4a), which can indicate that the time-series clusters are able to spatially group areas with higher species observations into distinct clusters. Especially cluster C6 encompasses relatively many high-quality pixels, with 50% of the highest quality areas (~ quality ‘9’) lying within this cluster (Fig. 4b). In the spatial clustering, there were no differences in the median landscape quality between clusters (Fig. 4a). All four clusters C2,C3,C6,C7 that show a distribution skewed towards higher landscape qualities show a higher AHA in the past. In Fig. 4b for the spatial clustering, Cluster C3 has a higher proportion of highest quality (‘9’) values, yet the median landscape quality is indistinct from the other clusters in the spatial clustering (Fig. 4a) indicating that most quality values in this cluster are below five.

The permutation tests on the cluster labels, identifying how often the cluster significantly overlaps or deters species occurrences, show that the time-series has more significant clusters (nine out of ten) than spatial clustering (four out of eight) at 99% confidence (Fig. 4c). Time-series clusters C1,C2,C3,C6,C7 and C10 hold more presences than expected from a random allocation of clusters, including the clusters with higher median landscape quality. Time series clusters C4,C5 and C9 have significantly lower number of species presences when compared to a random distribution of clusters, indicating that these areas significantly deter species from being present. These are also the clusters that are mainly located within cities and its suburban areas. Therefore it is notable that the oversampling near cities as mentioned in Rutishauser et al. 2023 is not a likely cause of bias in our results.

### Modeling species data

The AUC values and effect sizes from the GLMs using AHA or HMD as explanatory variables show clear differences across time steps and dispersal thresholds. The evolution of the AUC and effect sizes over time, following a somewhat unimodal curve with its peak at 1933, indicates the importance of the model with historical AHA values over the models with current data. The AUC values range from 0.53 to 0.59 across time steps with 1933 showing the highest. Additionally, when comparing HMD and AHA, we found the AUC values change from 0.51 to 0.59 indicating a notable advantage in the AHA model’s ability to discriminate between species presence-absence. The effect size that shows the increase in probability of occurrence of at least one indicator species per unit standard deviation of AHA also shows similar patterns over time and in its comparison to HMD. The effect size of the predictors for AHA is positive (>0.5) whereas for HMD is negative (<0.5). This implies that the higher the AHA or the lower the HMD, the higher the probability of species occurrence. However, the highest achieved AUC and effect size are still on the lower end (~ 0.6) implying poor discrimination and effect on species presence-absence. This implies that the AHA alone is not sufficient to fully explain the species presence-absence and must be used in conjunction with other variables defining species occurrence.

For the different dispersal scales, we can see that both the AUC and effect size of models using the data of 1933 is the highest for dispersal distance of 2000 m. For dispersal distances of 250 m and 500 m such an influence of the past is less evident, where for 250 m both the metric values increase over time, yet overall perform worse than the other dispersal scales. Additionally, both with HMD and AHA, using all the scales together (‘Allscales’) in a model provides a better AUC value. We conclude that despite the limitations of a simplified model, a positive correlation emerges between species occurrence probability and AHA value. This effect is most distinct from the historical time-step of 1933.

**Fig. 5:**
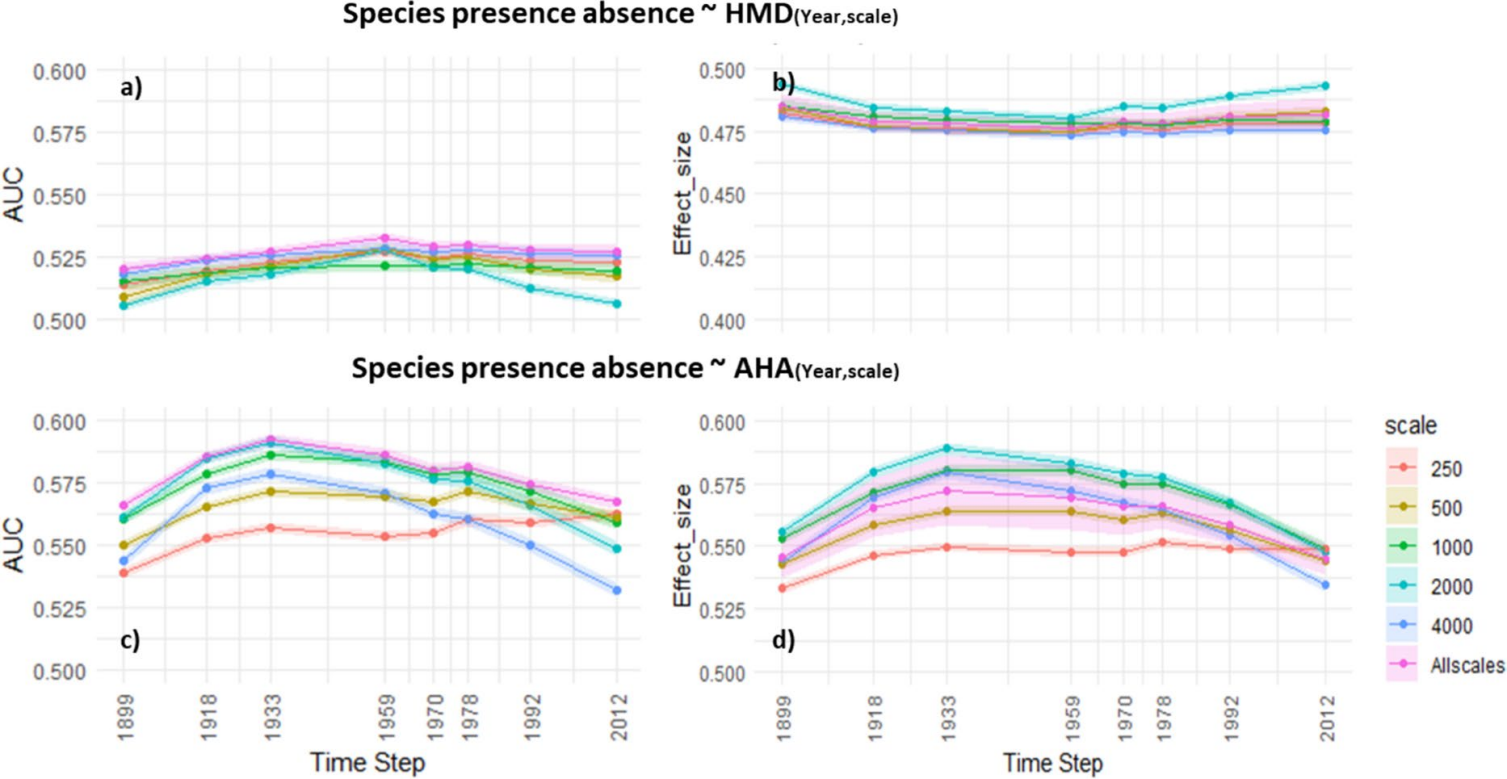
Shows the AUC values (**a**, **c**) and the Effect size (**b**, **d**) for the GLM models using HMD (top row) and AHA (bottom row) for different timesteps with contemporary species presence-absence data in the Swiss plateau. AUC indicates the ability of the model to discriminate species presence-absence, and the Effect size indicates the increment in probability of species occurrence (at least one indicator species) per standard deviation change in AHA or HMD. The error ribbon is based on different realisations of subsampling at 30% of the data

We also ran polynomial regressions (square terms) of the variables in the models and found similar AUC values, except for the ‘Allscales’ 2012 model that gained predictive power, while all individual scales of 2012 models performed worse (See Supplementary material S11). It is important to mention that the AHA of 2012 is not without any predictive power and performs better than HMD, as can also be seen in Fig. 5, yet the historical AUC of 1933 is still higher throughout, even in the polynomial versions. Finally, we also ran full models will all the datapoints without subsampling, and the results were similar (see Supplementary material table S14).

## Discussion

We find that the Amount of Habitat Available (AHA) as a proportion of reachable habitat within a dispersal distance threshold is a dimensionless ratio that can be used to quantify the impact of human-made development on species occurrences. We find this for a species group that prefers extensive agricultural landscape mosaics. We conducted a multi-scale clustering analysis on trajectories of AHA change over the 113-year period preceding 2012 on indicator species occurrences. We found that this method is able to identify areas of high occurrence of our chosen indicator species, with 50% of the highest-quality habitat areas concentrated within a single cluster. These areas with observations of expert curated list of indicator species signal landscapes of good contemporary habitat quality, however our results suggest that the occurrence of these species is better explained by models using historical information. These results thus underscore the importance of incorporating time-series data on landscape changes to effectively identify areas of good landscape quality. We reiterate here that our landscape quality data identifies areas with at least one expert‐curated indicator species rather than overall species richness. This approach is based on the idea that even a single indicator species—selected for its sensitivity to habitat conditions—signals a minimum threshold of habitat quality. Consequently, we validate our AHA metric on the occurrence of at least one key species, providing a reliable proxy for landscape quality, without attempting to infer overall species abundance or diversity.

The main result from our study is that historical habitat availability correlates better with contemporary species occurrences than current habitat availability. We found that the time-series clusters in which our chosen indicator species was most prevalent were the clusters that had the highest habitat availability until 1933, but not necessarily the highest habitat availability in more recent decades. Furthermore, we found that the probability of occurrence of our species group was best explained by habitat availability of 1933. These results suggest that there could be a strong temporal lag in species response to landscape changes for our chosen species group. This time-lagged response can indicate an extinction debt, which can be due to be paid off in the future (Kuussaari et al. 2009; Guardiola et al. 2013; Hughes et al. 2013). The 80 year time-lag since 1933, additionally can be indicative of the order of magnitude of the relaxation time, defined as the time taken by the species to reach a new equilibrium post perturbation or habitat turnover (Chen et al. 2023). Identifying the exact value of the extinction debt and the relaxation time is important (Lalechère et al. 2019), however, is beyond the purview of our study. Nevertheless, our findings are plausible, as time lagged responses to habitat change spanning several decades have been found for multiple other species in Europe, which can indicate a broader trend encompassing multiple species (Dullinger et al. 2013; Purschke et al. 2014; Liao et al. 2022; Scherreiks et al. 2022; Harisena et al. 2024). Therefore, for long term conservation of species information on time-lagged changes of habitat availability is important, and the analysis of only a snapshot of the landscape in time can confound inference (Essl et al. 2015a).

One reason of such a temporal lag could be, as stated by Martins & Pereira, (2017), that species extinctions in human modified areas are delayed if species are not very sensitive to human-made changes in the landscape. This can especially happen when the barriers to dispersal are not absolute, allowing species to continue to persist and (re)colonise other habitable areas long after barriers have been installed. It could be that the chosen indicator species have a relatively low sensitivity to human modification of the landscape and therefore can continue to persist in such landscapes (Miller et al. 2015). Therefore it is important to note here that this time lagged-responses can be a specific outcome for the chosen species group and that species that are more specialised and/or react strongly to human modification respond faster to landscape changes (Krauss et al. 2010; Ramírez-Delgado et al. 2022). For species with more specific habitat preferences, resistance surfaces would need to be carefully modelled by including multiple aspects of their habitat requirements such as climate, elevation, vegetation cover etc. (Trainor et al. 2013; Zeller et al. 2024). However, for a generalist indicator species group that prefers mosaic landscapes, our method can provide viable inferences.

We further found that temporal trajectories, as captured in the time-series clusters, provide essential context for understanding landscape transformations, capturing both the landscape’s initial conditions and the rate and pattern of change over time. Incorporating temporal information, which captures a wider range of historical habitat availability, enhances contemporary data by grouping trajectories where variability has decreased. This process refines spatial clusters that have less variation, making the resulting spatio-temporal clusters more informative. In our study, we identified distinct clusters with different trajectories of AHA change, which excel in identifying regions of high empirical species observations when compared to spatial clustering using contemporary spatial data alone. We also identified clusters with significantly less species occurrences than expected by chance using the time-series trajectories, corresponding to urban and sub-urban areas. This serendipitously shows that the analysis of our metric is not conflated with biases in observation towards areas of higher human activity, as is a common bias in species observation datasets. However, the exact parameters of the trajectories that contribute to species responses, beyond the high historical AHA values, still require further research. Studies have identified higher extinction debts in habitat patches that have declined in area in the past when compared to stable ones (Guardiola et al. 2013). It can be then that the interplay between initial landscape conditions, rates of change across different timescales and species behavioural adaptations may alter extinction debts and species relaxation times, leading to cluster-specific variations in species responses (Jackson & Sax 2010; Wearn et al. 2012; Essl et al. 2015b).

The metric we introduce in this study, the AHA, outperforms the HMD in modelling indicator species presence-absence, despite both being derived from the same landcover data. The HMD represents the current best practice in landscape ecology, summarizing the landscape using a moving window analysis with a fixed shape (Eigenbrod et al. 2008; Belote et al. 2022). However, the AHA introduces a significant innovation by moving beyond this fixed-shape paradigm. Unlike the HMD, the AHA employs a variable mesh shape and size to the moving windows, tailored to the dispersal traits of the species and the surrounding landscape features. Importantly, the AHA adapts its analysis to species-specific traits, such as their capacity to cross barriers and their dispersal abilities (Krosby et al. 2010). Thus, it models habitat availability from the perspective of species moving within a landscape, capturing process-based functional responses that purely pattern-based analyses like the HMD cannot detect.

An explanation for the fact that AHA was a better predictor for species occurrences than HMD, is that the AHA responds to both the densities and configurations of roads and buildings, whereas HMD only assesses densities, and thus may not capture information on certain local landscape variabilities (Fischer & Lindenmayer 2007; Eigenbrod et al. 2008). For example, AHA is low for a species dispersing from a location surrounded by strong barriers and will be affected neither by open land nor dense settlements beyond these immediate barriers. However, the HMD for both the cases (open land vs dense settlement), will vary, capturing aggregate information not relevant to species dispersal. Thus, AHA efficiently captures reachable habitats only in the species immediate vicinity based on both the species dispersal ability and the local configuration of the human-modified landscape. It is also interesting to note that the HMD does not perform much better in the past than its 2012 values. This can indicate that the processes identified in the AHA that are not captured in HMD might contribute to time-lagged species responses, for e.g. species ability to find refuge in the available habitat in the vicinity, and thus persisting longer in modified landscapes (Hylander & Ehrlén, 2013).

Even though AHA was the only explanatory variable for the presence or absence of the species, the model still managed to explain variation in occurrences, albeit with a fairly poor discrimination power marginally reaching an AUC of 0.6 in 1933. Notably, key factors such as climate, vegetation, and other environmental variables, known to influence species distributions (Guisan & Zimmermann 2000), were not included. AHA should thus be viewed as a complementary variable, capturing how dispersal constrains habitat availability for mobile species. Additionally, we include historical landscape legacies using this metric that is an uncommon yet important variable to be incorporated in modelling species occurrences (Zimmermann et al. 2010). Even within the modelling of dispersal based processes our formulation of the AHA does not include other movement traits like perceptual ranges and steppingstone movement along with topographic elements like elevation that may also play a role in structuring populations (Rocha et al. 2021). Such processes could be included with individual based models that explicitly include other mechanisms for species dispersal (Unnithan Kumar et al. 2022; Fletcher et al. 2023; Tao et al. 2024). However, whereas such individual based models may bring more realism and variability in the dispersal patterns, they are also computationally challenging to implement across large areas and for multiple time-steps, as we did with the AHA.

**Fig. 6:**
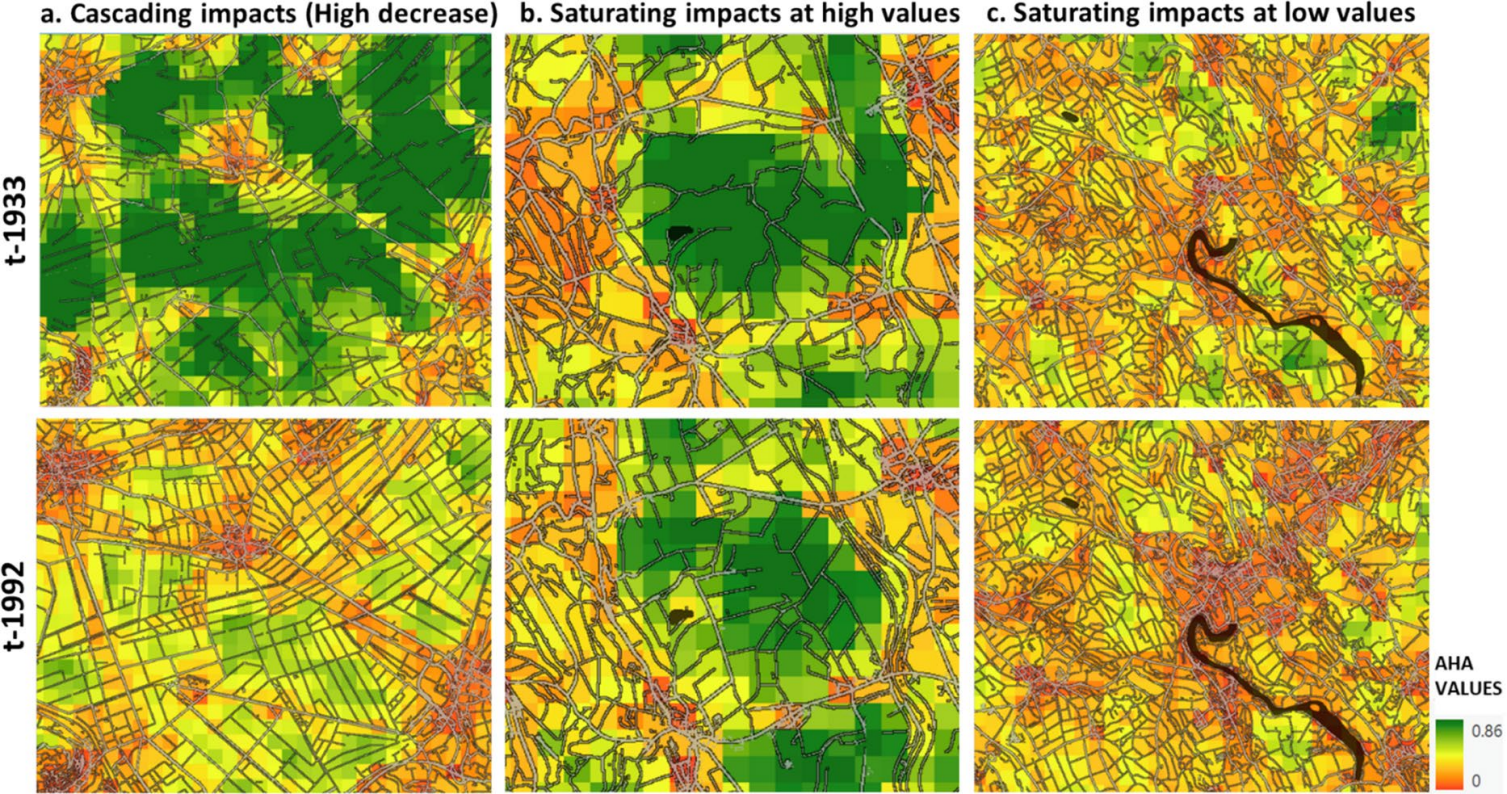
Shows three different patterns of temporal change in AHA for a maximum dispersal distance of 1000 m, between 1933 (top-row) and 1992 (bottom-row) with respect to the input road and building patterns; for example regions from different clusters; **a** visualizes the spatial patterns for high decrease (cascading) in AHA in cluster C6; **b** visualizes spatial patterns for high and stable (Absorbing) AHA in cluster C2; and **c** visualizes the spatial patterns for low and stable (Saturating) AHA in cluster C5. Each pixel is 250 by 250 m in dimension

In AHA estimations, the combined influence of density and configuration allows temporal landscape changes to aggregate in various ways. By visually examining the different trajectories, we have identified three main patterns in this: *cascading* (exponential decline), *saturating at high values* (remaining stable at a high level), and *saturating at low* values (remaining stable at a low level) impacts of land cover change over time (Fig. 6).

In Fig. 6a, we see a region with mainly time-series cluster C6 pixels showing a sharp AHA decline after 1933. Interestingly, while HMD increases in this region, it doesn’t fully account for the steep AHA drop. This decrease is likely due to the cumulative impact of road development and evolving road configurations. Until 1933, road edges were more open, but they became increasingly closed over time, amplifying configurational effects and driving a cascading AHA decline. In contrast, Fig. 6b mainly consists of pixels with time series cluster C2 and shows minimal AHA change despite rising HMD. Here, AHA remains high, yet future cascading losses remain a risk. In Fig. 6c most pixels are of time-series cluster C5 with AHA remaining consistently low even as HMD rises, suggesting a saturated response where increased human modification no longer affects AHA. These patterns underscore how AHA trajectories vary with landcover changes and initial landscape conditions, revealing multiple relationships between AHA, HMD, and configuration. Understanding such time-lagged dynamics is essential for assessing extinction debt risks and planning landscapes that can still support biodiversity despite high human modification (Essl et al. 2015a).

Our findings highlight the value of incorporating long-term, multi-scale analyses to understand biodiversity’s dependence on historical landscape configurations (Pan et al. 2022). The AHA metric captures immediate habitat availability for generalist species with varying dispersal abilities, accounting for barriers such as roads and buildings. We demonstrate that historical cartographic sources can provide useful data for modelling species occurrences, and future digitisation of archived maps could further enhance such efforts. The temporal trajectories in our dataset rely on curated inputs from multiple sources—the Siegfried maps, Old National Maps, and the contemporary Swiss TLM. Slight aberrations appear around 1959 and 2012 due to dataset transitions, but as these are consistent across clusters, the clustering output remains robust. Nonetheless, future studies should aim for consistent temporal topologies to ensure smoother transitions and greater comparability over time. Our results also emphasize the utility of a cross-scale approach (“Allscale”) for assessing habitat availability when the optimal dispersal threshold is uncertain. While a 2000 m threshold proved informative, combining multiple scales was a flexible and effective alternative. In species-agnostic contexts, where defining a single spatio-temporal scale is difficult, multi-scale approaches such as ours offer valuable insights. The AHA metric, paired with this cross-scale framework, presents a practical, adaptable tool for linking historical landscape change to current biodiversity patterns, supporting conservation in complex, human-modified environments.

## Conclusion

Our study emphasizes the importance of identifying time-series of trajectories of habitat availability change to assess the current occurrence of a generalist indicator species that prefer mosaic cultivated landscapes and meadows. It introduces a novel metric, the Amount of Available Habitat (AHA) that uses a species-oriented perspective to define the amount of habitat reachable by the species within a highly human-modified landscape that poses resistance to dispersal. By using time-series clustering of habitat availability (AHA) over the past century in the Swiss plateau, the study identifies distinct trajectories of landscape change that correlate more strongly with species presence than static, spatial approaches. The findings show that historical habitat availability, especially around 1933, better predicts the presence of indicator species, suggesting the existence of an extinction debt. Importantly, AHA’s sensitivity to both the density and configuration of human-modified landscapes provides critical insights that static, pattern-based metrics like HMD cannot fully capture. These findings underscore the need for long-term, multi-scale analyses that include both spatial and temporal dimensions to inform conservation strategies, especially in human modified landscapes.

## Supplementary Information

Below is the link to the electronic supplementary material.

Supplementary file1 (DOCX 8306 KB)

## Data Availability

The datasets generated during and/or analysed during the current study are available from the corresponding author on reasonable request. The code used to calculate the Amount of Habitat Available (AHA) metric can be found here: 10.5281/zenodo.15114363.
