# Supplementary material for: A novel method to assess spatio-temporal habitat availability for a generalist indicator species group in human-modified landscapes
Source: Landsc Ecol. 2025 May 17;40(6):103. doi: 10.1007/s10980-025-02124-x (PMC12085322; doi:10.1007/s10980-025-02124-x)
Supplement: Supplementary file 1 — Supplementary file1 (DOCX 8306 KB) [file 10980_2025_2124_MOESM1_ESM.docx]

# Supplementary material

**S1. Correction of road segmentation outputs**

Step 1: Threshold original data to reduce initial artefacts. The threshold was set to >0.2 by iteratively checking at what point sufficient information without noise was captured.


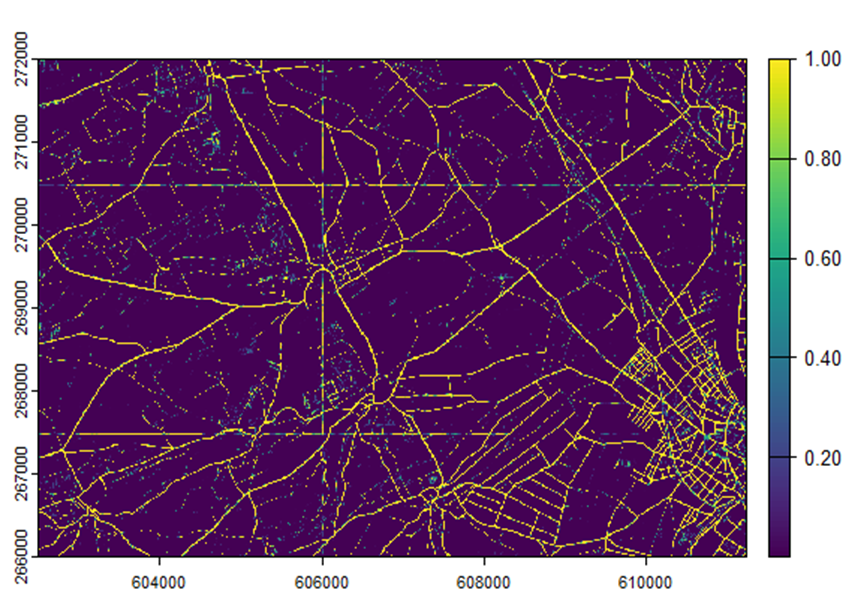


Step 2: Separating orthogonal gridlines and larger road data. After thresholding some grid like structures remained which needed to be removed. This is done by aggregating the input data vertically (fact(1,4)) and horizontally (fact(4,1)) such that the orthogonal gridlines will always have higher values than the organic ones and are thus detected. The detection is undertaken by making polygons and dropping smaller polygons, leaving the larger orthogonal polygons intact.


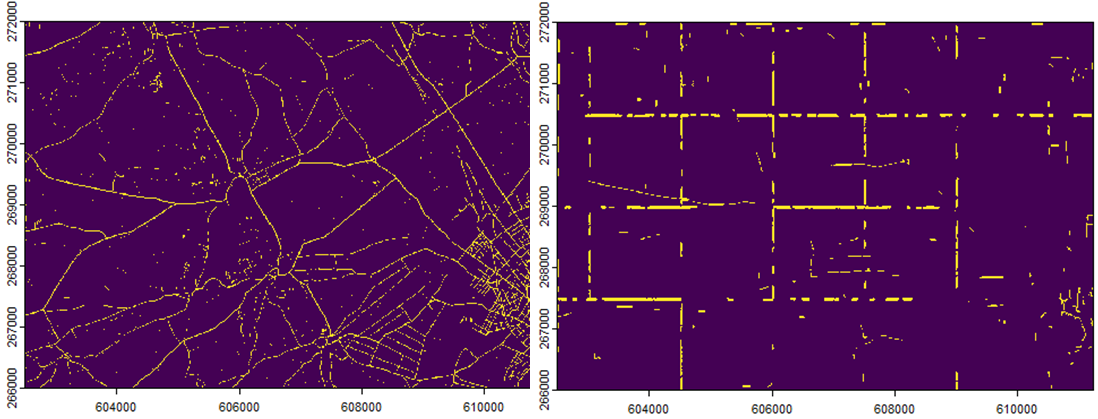


Step 3: The grids are then subtracted from original raster to get the actual roads out. Additionally the roads detected above are again aggregated and re-added to the original to strengthen road signal in the original. But output still has a lot of artefacts. This is removed by again converted to polygons, buffering the polygons and merging overlapping ones to connect road segments and then removing polygons at a higher area threshold. Then de-buffering the data, masking the resulting polygonal area from the original raster.


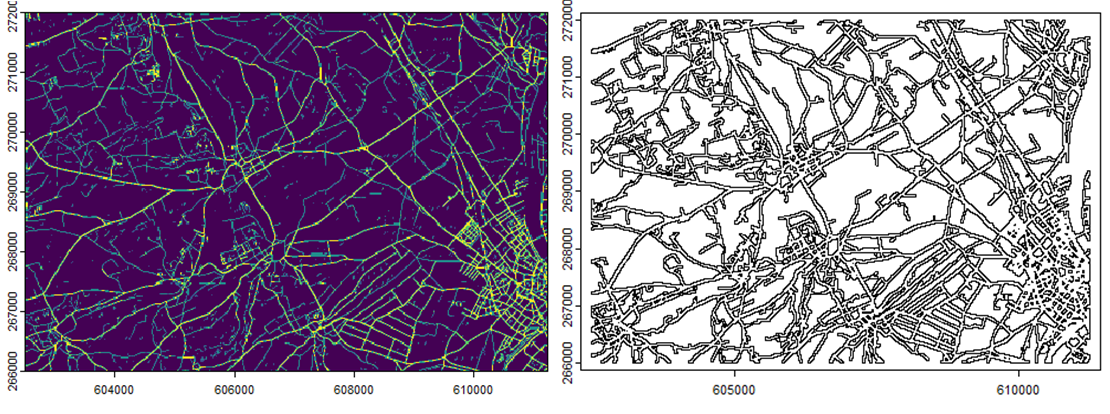


Step 4: The original raster masked using the large connected road polygon is smoothened using a gaussian focal function and then classified into the final road output.


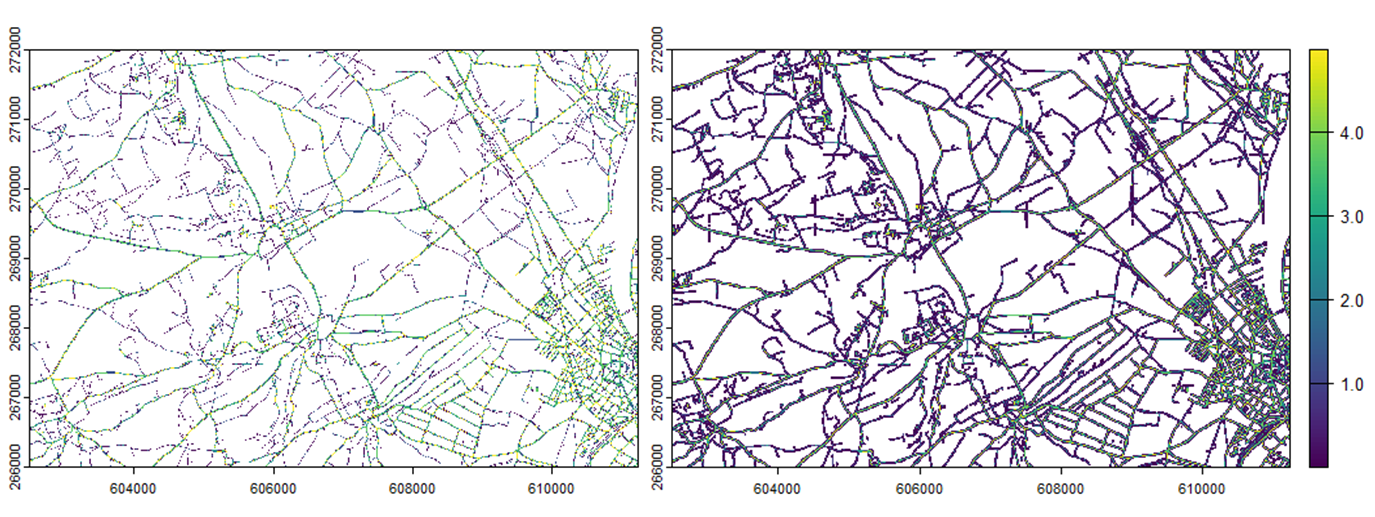


Step 5: Post clean-up certain artefacts remain, yet with much lower frequency. This was repeated for all the map sheets across the Swiss plateau for eight time-steps.

**S2. Table showing the mapping between Swiss TLM 2012 road data classes and road widths from the past historical maps**

| SwissTLM 2012_road_classes | Width REF |
| --- | --- |
| 0 | 60 |
| 1 | 60 |
| 2 | 60 |
| 3 | 60 |
| 4 | 60 |
| 5 | 60 |
| 6 | 60 |
| 7 | 60 |
| 8 | 15 |
| 9 | 40 |
| 10 | 40 |
| 11 | 40 |
| 12 | 15 |
| 14 | 15 |
| 15 | 20 |
| 16 | 15 |
| 17 | 15 |
| 18 | 15 |
| 19 | 15 |
| 21 | 60 |

**S3. Details of the chosen indicator species list**


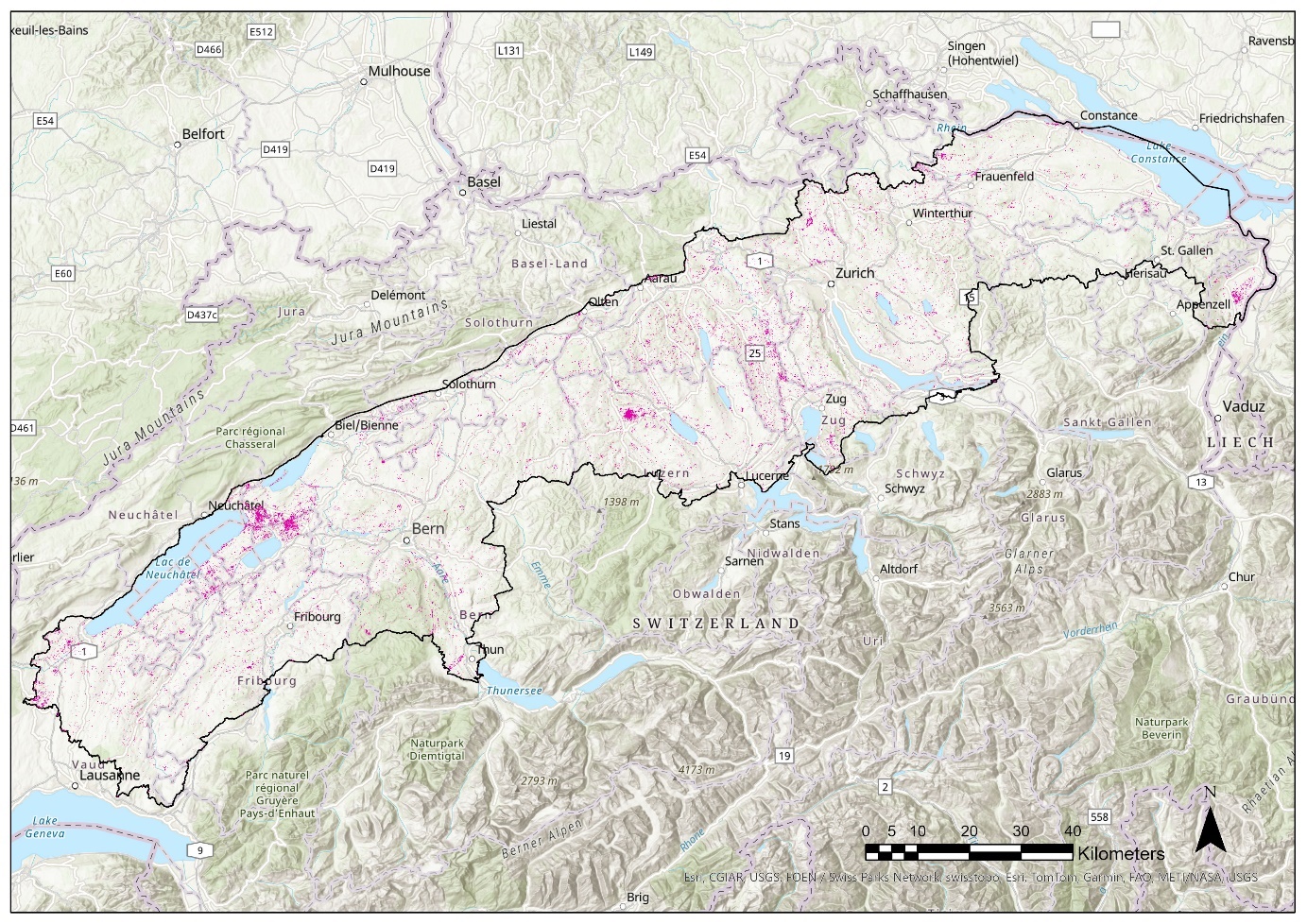


Map showing the Swiss plateau (black boundary) and the spatial distribution of the pixels with at least 1 indicator species (pink pixels).

The Guild 25 encompasses 54 indicator species, as curated by experts, that inhabit mosaics of arable land and meadows in Switzerland. The species list with TAXON names are given below:

| **GROUP** | **TAXON_ID** | **TAXON_NAME** |
| --- | --- | --- |
| AMPH | 70100 | Salamandra atra Laurenti, 1768 |
| AVES | 4970 | Anthus trivialis |
| AVES | 3130 | Athene noctua (Scopoli, 1769) |
| AVES | 5370 | Carduelis cannabina (Linnaeus, 1758) |
| AVES | 1350 | Circus pygargus |
| AVES | 1610 | Coturnix coturnix |
| AVES | 5570 | Emberiza calandra Linnaeus, 1758 |
| AVES | 5640 | Emberiza cirlus Linnaeus, 1766 |
| AVES | 5580 | Emberiza citrinella |
| AVES | 5670 | Emberiza hortulana Linnaeus, 1758 |
| AVES | 1480 | Falco tinnunculus Linnaeus, 1758 |
| AVES | 3370 | Jynx torquilla Linnaeus, 1758 |
| AVES | 5160 | Lanius collurio |
| AVES | 5140 | Lanius senator Linnaeus, 1758 |
| AVES | 3560 | Lullula arborea (Linnaeus, 1758) |
| AVES | 3080 | Otus scops (Linnaeus, 1758) |
| AVES | 1600 | Perdix perdix (Linnaeus, 1758) |
| AVES | 4070 | Phoenicurus phoenicurus (Linnaeus, 1758) |
| AVES | 3380 | Picus viridis |
| AVES | 4090 | Saxicola rubetra (Linnaeus, 1758) |
| AVES | 4100 | Saxicola rubicola (Linnaeus, 1766) |
| AVES | 3000 | Streptopelia turtur (Linnaeus, 1758) |
| AVES | 4600 | Sylvia borin (Boddaert, 1783) |
| AVES | 4610 | Sylvia communis Latham, 1787 |
| AVES | 3070 | Tyto alba (Scopoli, 1769) |
| AVES | 3360 | Upupa epops Linnaeus, 1758 |
| AVES | 1850 | Vanellus vanellus (Linnaeus, 1758) |
| CHIR | 70715 | Myotis daubentonii |
| CHIR | 70719 | Myotis mystacinus |
| CHIR | 70722 | Myotis nattereri |
| CHIR | 70732 | Plecotus auritus |
| CHIR | 70701 | Rhinolophus hipposideros |
| MAMM | 70767 | Crocidura leucodon (Hermann, 1780) |
| MAMM | 70769 | Crocidura suaveolens (Pallas, 1811) |
| MAMM | 70800 | Lepus europaeus Pallas, 1778 |
| MAMM | 70749 | Mustela erminea |
| MAMM | 70751 | Mustela nivalis Linnaeus, 1766 s.l. |
| MAMM | 70773 | Sorex araneus |
| MAMM | 70774 | Sorex coronatus |
| REPT | 70156 | Anguis fragilis |
| REPT | 70171 | Anguis veronensis |
| REPT | 70158 | Coronella austriaca |
| REPT | 70157 | Hierophis viridiflavus |
| REPT | 70151 | Lacerta agilis |
| REPT | 70152 | Lacerta bilineata |
| REPT | 70174 | Natrix helvetica |
| REPT | 70161 | Natrix natrix |
| REPT | 70154 | Podarcis muralis |
| REPT | 70163 | Vipera aspis |
| REPT | 70164 | Vipera berus |
| REPT | 70159 | Zamenis longissimus |
| REPT | 70153 | Zootoca vivipara |

Further details for this can be found here: <https://www.infospecies.ch/fr/projets/infrastructure-ecologique.html#listen>

**S4: Resistance values assigned to landcover information based on specific scenarios of relative cost indicating effort for movement and ranking of barriers in the landscape**

| **LC** | **Rank** | **Scen1** | **Scen2** | **Scen3** | **Scen4** | **Scen5** |
| --- | --- | --- | --- | --- | --- | --- |
| Road | 2.5 | 42 | 35 | 25 | 12 | 3 |
| building | 3 | 50 | 50 | 50 | 50 | 50 |
| Natural | 1 | 1 | 1 | 1 | 1 | 1 |
| Lake | 2 | 33 | 22 | 10 | 20 | 1 |

**S5. Optimum number of clusters using elbow method:**


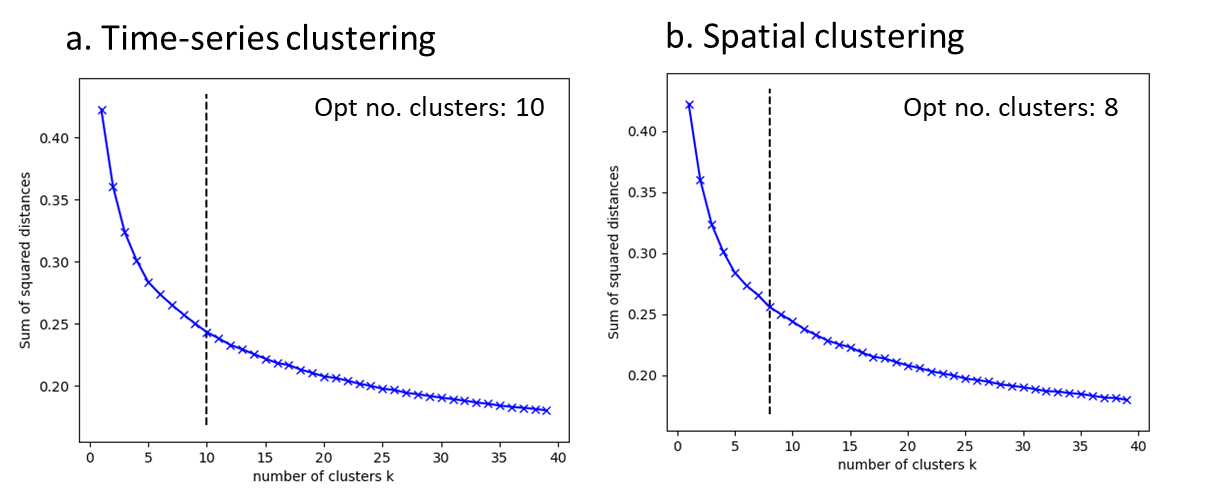


**S6. Table showing the time-series cluster means of AHA for different spatial scales and time-steps**

| **Scale** | **Cluster** | **Mean_1899** | **1918** | **1933** | **1959** | **1971** | **1978** | **1992** | **2012** |
| --- | --- | --- | --- | --- | --- | --- | --- | --- | --- |
| 250 | 1 | 0.21 | 0.19 | 0.20 | 0.31 | 0.30 | 0.31 | 0.31 | 0.39 |
| 500 | 1 | 0.20 | 0.18 | 0.19 | 0.23 | 0.22 | 0.22 | 0.21 | 0.26 |
| 1000 | 1 | 0.21 | 0.19 | 0.19 | 0.21 | 0.20 | 0.20 | 0.19 | 0.21 |
| 2000 | 1 | 0.21 | 0.20 | 0.20 | 0.21 | 0.20 | 0.19 | 0.19 | 0.20 |
| 4000 | 1 | 0.23 | 0.21 | 0.21 | 0.22 | 0.20 | 0.20 | 0.19 | 0.19 |
| 250 | 2 | 0.69 | 0.66 | 0.65 | 0.66 | 0.63 | 0.63 | 0.60 | 0.61 |
| 500 | 2 | 0.49 | 0.45 | 0.44 | 0.43 | 0.41 | 0.40 | 0.38 | 0.39 |
| 1000 | 2 | 0.33 | 0.31 | 0.30 | 0.30 | 0.28 | 0.28 | 0.26 | 0.26 |
| 2000 | 2 | 0.29 | 0.26 | 0.25 | 0.26 | 0.24 | 0.23 | 0.22 | 0.21 |
| 4000 | 2 | 0.28 | 0.25 | 0.24 | 0.24 | 0.23 | 0.22 | 0.21 | 0.20 |
| 250 | 3 | 0.50 | 0.45 | 0.45 | 0.47 | 0.45 | 0.45 | 0.43 | 0.46 |
| 500 | 3 | 0.32 | 0.28 | 0.28 | 0.28 | 0.27 | 0.26 | 0.25 | 0.26 |
| 1000 | 3 | 0.24 | 0.22 | 0.21 | 0.22 | 0.21 | 0.20 | 0.19 | 0.19 |
| 2000 | 3 | 0.23 | 0.21 | 0.20 | 0.21 | 0.19 | 0.19 | 0.18 | 0.18 |
| 4000 | 3 | 0.24 | 0.21 | 0.21 | 0.21 | 0.20 | 0.19 | 0.18 | 0.18 |
| 250 | 4 | 0.36 | 0.28 | 0.23 | 0.13 | 0.09 | 0.08 | 0.07 | 0.13 |
| 500 | 4 | 0.24 | 0.19 | 0.16 | 0.11 | 0.09 | 0.08 | 0.07 | 0.10 |
| 1000 | 4 | 0.20 | 0.17 | 0.15 | 0.12 | 0.09 | 0.09 | 0.08 | 0.10 |
| 2000 | 4 | 0.20 | 0.17 | 0.16 | 0.13 | 0.11 | 0.11 | 0.10 | 0.10 |
| 4000 | 4 | 0.22 | 0.19 | 0.17 | 0.15 | 0.13 | 0.13 | 0.12 | 0.12 |
| 250 | 5 | 0.09 | 0.06 | 0.06 | 0.06 | 0.04 | 0.04 | 0.03 | 0.04 |
| 500 | 5 | 0.08 | 0.06 | 0.06 | 0.06 | 0.04 | 0.04 | 0.04 | 0.04 |
| 1000 | 5 | 0.09 | 0.07 | 0.07 | 0.07 | 0.05 | 0.05 | 0.04 | 0.04 |
| 2000 | 5 | 0.12 | 0.10 | 0.09 | 0.09 | 0.07 | 0.07 | 0.06 | 0.06 |
| 4000 | 5 | 0.16 | 0.13 | 0.13 | 0.12 | 0.10 | 0.10 | 0.09 | 0.08 |
| 250 | 6 | 0.75 | 0.70 | 0.62 | 0.38 | 0.32 | 0.30 | 0.27 | 0.38 |
| 500 | 6 | 0.58 | 0.52 | 0.45 | 0.28 | 0.23 | 0.22 | 0.20 | 0.25 |
| 1000 | 6 | 0.42 | 0.37 | 0.33 | 0.24 | 0.21 | 0.20 | 0.18 | 0.21 |
| 2000 | 6 | 0.34 | 0.30 | 0.27 | 0.22 | 0.20 | 0.18 | 0.17 | 0.18 |
| 4000 | 6 | 0.30 | 0.27 | 0.25 | 0.22 | 0.19 | 0.19 | 0.17 | 0.18 |
| 250 | 7 | 0.55 | 0.52 | 0.46 | 0.20 | 0.16 | 0.15 | 0.14 | 0.29 |
| 500 | 7 | 0.38 | 0.36 | 0.31 | 0.17 | 0.14 | 0.14 | 0.13 | 0.21 |
| 1000 | 7 | 0.29 | 0.27 | 0.24 | 0.17 | 0.14 | 0.14 | 0.13 | 0.17 |
| 2000 | 7 | 0.26 | 0.24 | 0.22 | 0.17 | 0.15 | 0.15 | 0.14 | 0.16 |
| 4000 | 7 | 0.25 | 0.23 | 0.22 | 0.18 | 0.16 | 0.16 | 0.15 | 0.16 |
| 250 | 8 | 0.37 | 0.33 | 0.32 | 0.32 | 0.29 | 0.27 | 0.24 | 0.25 |
| 500 | 8 | 0.22 | 0.19 | 0.19 | 0.18 | 0.16 | 0.15 | 0.14 | 0.14 |
| 1000 | 8 | 0.18 | 0.16 | 0.16 | 0.16 | 0.14 | 0.13 | 0.12 | 0.11 |
| 2000 | 8 | 0.19 | 0.17 | 0.16 | 0.16 | 0.14 | 0.14 | 0.13 | 0.12 |
| 4000 | 8 | 0.21 | 0.19 | 0.18 | 0.18 | 0.16 | 0.15 | 0.14 | 0.13 |
| 250 | 9 | 0.73 | 0.25 | 0.19 | 0.19 | 0.17 | 0.16 | 0.16 | 0.23 |
| 500 | 9 | 0.58 | 0.22 | 0.18 | 0.16 | 0.15 | 0.14 | 0.13 | 0.17 |
| 1000 | 9 | 0.43 | 0.21 | 0.18 | 0.17 | 0.15 | 0.14 | 0.13 | 0.16 |
| 2000 | 9 | 0.35 | 0.20 | 0.18 | 0.17 | 0.16 | 0.15 | 0.14 | 0.16 |
| 4000 | 9 | 0.32 | 0.21 | 0.19 | 0.18 | 0.17 | 0.16 | 0.15 | 0.16 |
| 250 | 10 | 0.10 | 0.09 | 0.10 | 0.13 | 0.12 | 0.12 | 0.12 | 0.18 |
| 500 | 10 | 0.14 | 0.12 | 0.12 | 0.13 | 0.12 | 0.12 | 0.11 | 0.15 |
| 1000 | 10 | 0.16 | 0.14 | 0.14 | 0.15 | 0.13 | 0.13 | 0.12 | 0.14 |
| 2000 | 10 | 0.18 | 0.17 | 0.16 | 0.17 | 0.15 | 0.15 | 0.14 | 0.15 |
| 4000 | 10 | 0.21 | 0.19 | 0.18 | 0.19 | 0.17 | 0.16 | 0.16 | 0.16 |

**S7. Spatial clustering map**


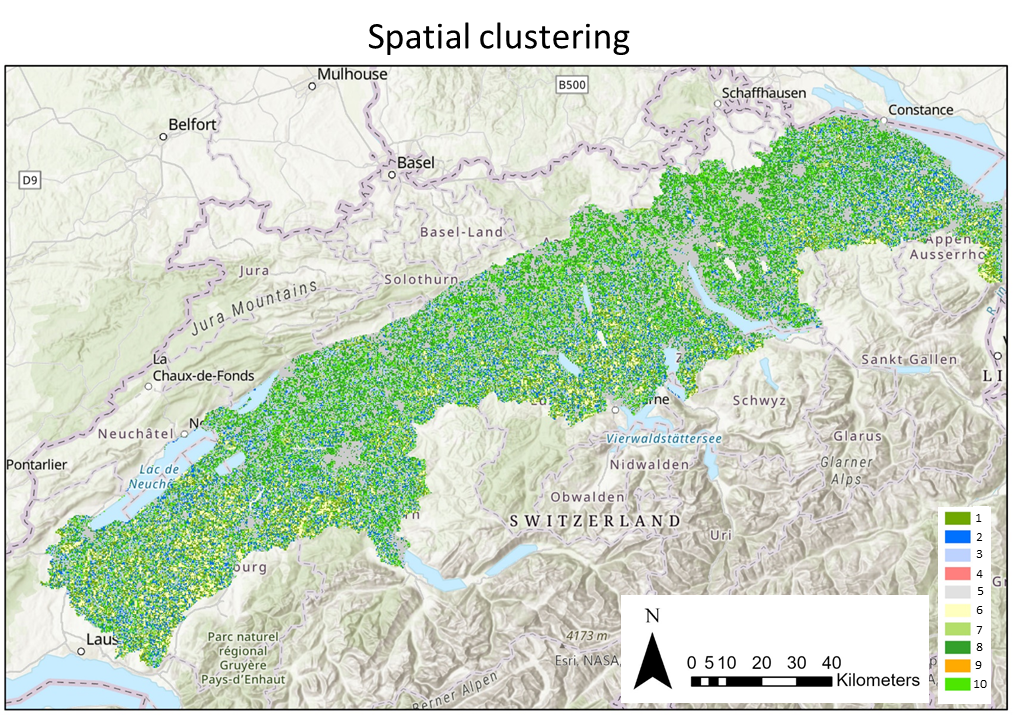


**S8. Available Habitat Amount (AHA) values**

Fig. shows the spatial distribution of the calculated Available Habitat Amount (AHA) values per 250 by 250 m pixel. We map this for a zoomed in region within the Zurich canton in the Swiss plateau. Fig.4. shows results for all dispersal distances for three time-steps i.e. 1899, 1949 and 1992. The values of accessibility fall between 0 and 1 since it denotes the proportional area accessible for the species given a maximum dispersal ability in a homogenous landscape. Fig.4. shows that over time the proportional accessibility has decreased considerably. Also, as you look at the maps for the higher dispersal, the range of accessibility values decrease since species tend to encounter higher barriers cumulatively at larger distances. Additionally, at higher dispersal distances, since more information form the surroundings is included to calculate the per-pixel accessibility, the maps appear smoothened over space.


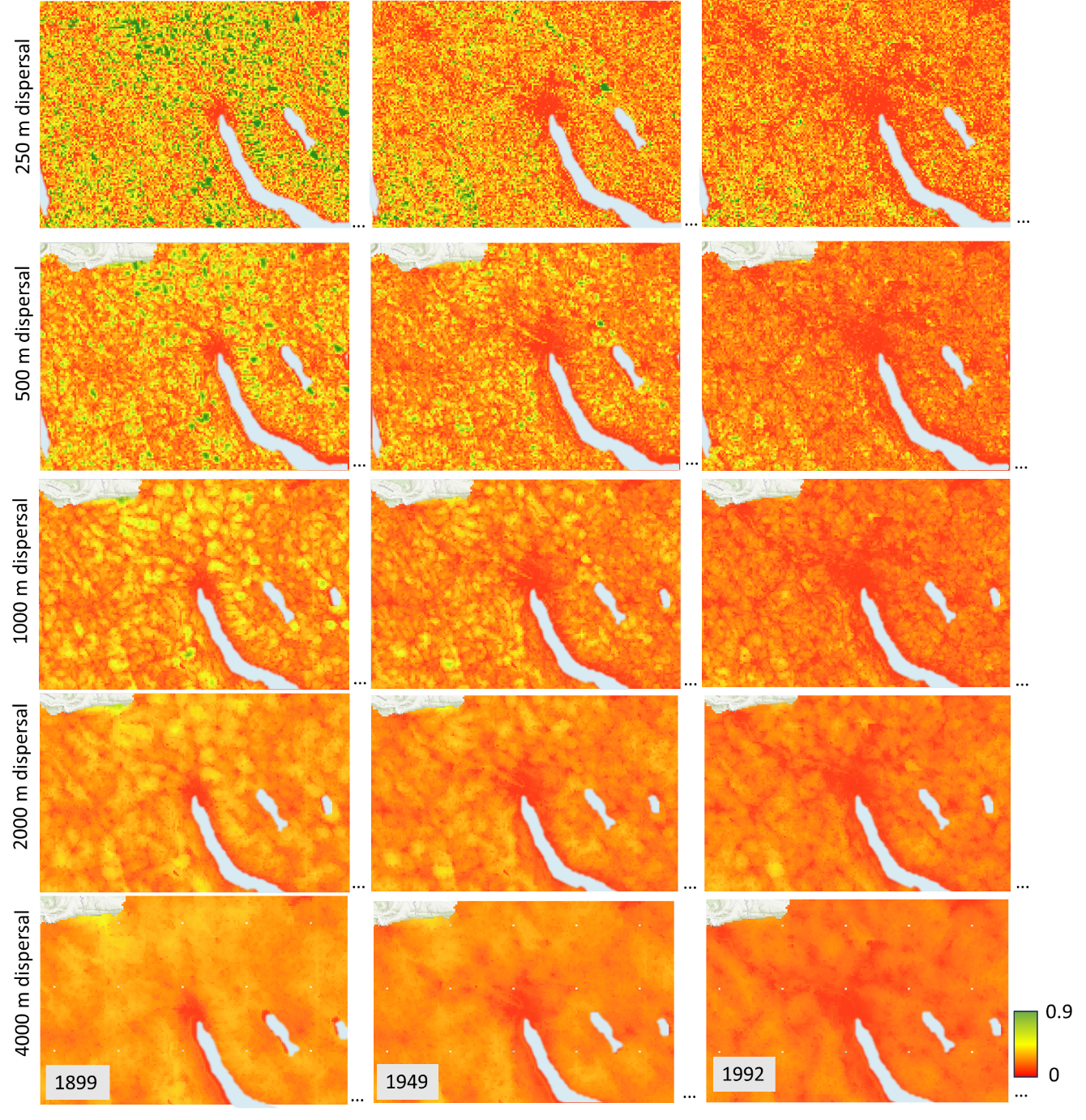


Accessibility values calculated for the entire Swiss plateau, but limited to a zoomed in area in the Zurich canton for visualization purposes. The rows represent the different dispersal distances and the columns represent different time-steps.

**S9. Confusion matrix between labels of time-series and spatial clustering methods**


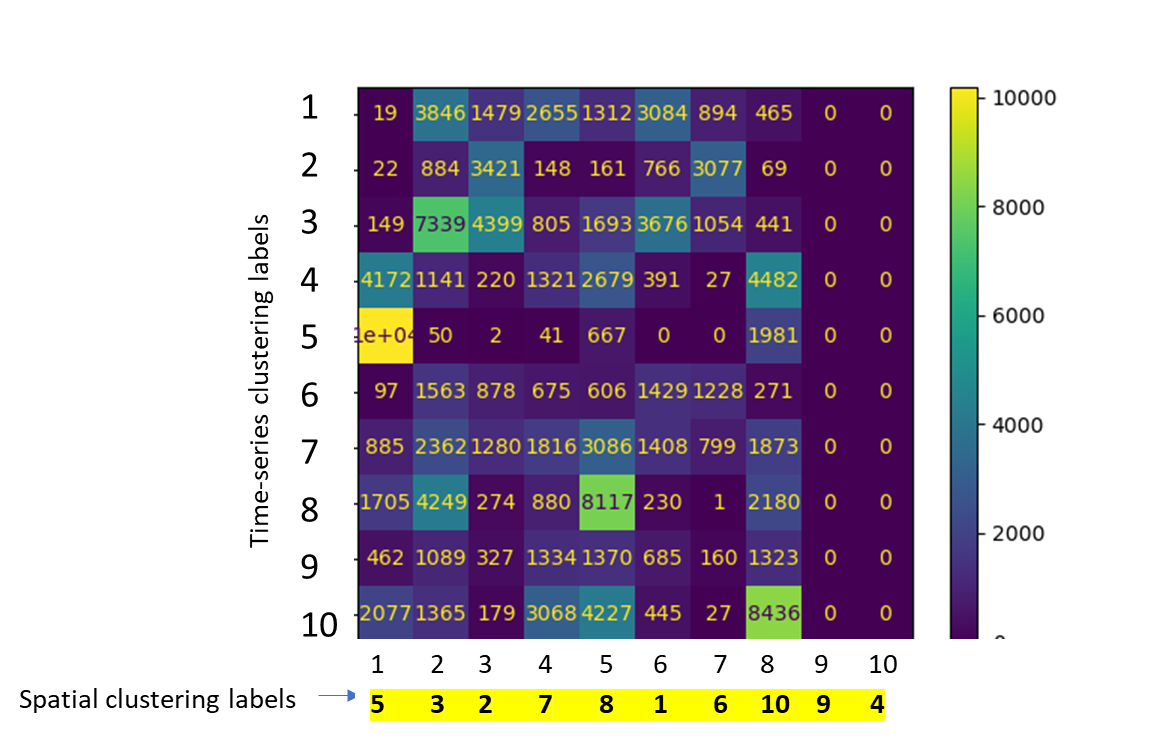


**S10. GLM model assumptions and details**

The dataset contains 133693 data points, each indicating a 250 by 250 m pixel in the Swiss plateau. Of this 27083 (~20%) are identified as areas of landscape quality i.e. having atleast 1 indicator species occurring.

|  | **Time_step** | **scale** | **T_uniform** | **T_dispersion** | **T_outliers** |
| --- | --- | --- | --- | --- | --- |
| 1 | 2012 | AllScales | 0.696294185 | 0.788 | 0.391082362 |
| 2 | 2012 | 250 | 0.879559391 | 0.864 | 0.358144298 |
| 3 | 2012 | 500 | 0.838779299 | 0.828 | 0.391082362 |
| 4 | 2012 | 1000 | 0.482055532 | 0.774 | 0.270185619 |
| 5 | 2012 | 2000 | 0.277792835 | 0.768 | 0.244477706 |
| 6 | 2012 | 4000 | 0.581574747 | 0.864 | 0.177812899 |
| 7 | 1992 | AllScales | 0.787676285 | 0.798 | 0.32701008 |
| 8 | 1992 | 250 | 0.962190458 | 0.94 | 0.391082362 |
| 9 | 1992 | 500 | 0.962388409 | 0.884 | 0.391082362 |
| 10 | 1992 | 1000 | 0.831432642 | 0.786 | 0.32701008 |
| 11 | 1992 | 2000 | 0.513178964 | 0.766 | 0.358144298 |
| 12 | 1992 | 4000 | 0.43458779 | 0.84 | 0.244477706 |
| 13 | 1978 | AllScales | 0.76459186 | 0.77 | 0.358144298 |
| 14 | 1978 | 250 | 0.958886643 | 0.96 | 0.425798097 |
| 15 | 1978 | 500 | 0.977304547 | 0.868 | 0.391082362 |
| 16 | 1978 | 1000 | 0.901030909 | 0.772 | 0.270185619 |
| 17 | 1978 | 2000 | 0.691009435 | 0.768 | 0.32701008 |
| 18 | 1978 | 4000 | 0.749900404 | 0.826 | 0.220540145 |
| 19 | 1970 | AllScales | 0.770256072 | 0.784 | 0.425798097 |
| 20 | 1970 | 250 | 0.971369838 | 0.976 | 0.297691291 |
| 21 | 1970 | 500 | 0.967708371 | 0.894 | 0.391082362 |
| 22 | 1970 | 1000 | 0.877077529 | 0.804 | 0.358144298 |
| 23 | 1970 | 2000 | 0.823668182 | 0.804 | 0.32701008 |
| 24 | 1970 | 4000 | 0.899645674 | 0.846 | 0.297691291 |
| 25 | 1959 | AllScales | 0.801419285 | 0.862 | 0.425798097 |
| 26 | 1959 | 250 | 0.966156173 | 0.976 | 0.32701008 |
| 27 | 1959 | 500 | 0.917960501 | 0.932 | 0.462250233 |
| 28 | 1959 | 1000 | 0.753492247 | 0.878 | 0.358144298 |
| 29 | 1959 | 2000 | 0.912975103 | 0.862 | 0.391082362 |
| 30 | 1959 | 4000 | 0.774062019 | 0.896 | 0.358144298 |
| 31 | 1933 | AllScales | 0.673423013 | 0.702 | 0.177812899 |
| 32 | 1933 | 250 | 0.907040877 | 0.996 | 0.462250233 |
| 33 | 1933 | 500 | 0.9116324 | 0.94 | 0.270185619 |
| 34 | 1933 | 1000 | 0.85291374 | 0.806 | 0.244477706 |
| 35 | 1933 | 2000 | 0.648841928 | 0.722 | 0.198334582 |
| 36 | 1933 | 4000 | 0.841638046 | 0.826 | 0.391082362 |
| 37 | 1918 | AllScales | 0.632001773 | 0.672 | 0.244477706 |
| 38 | 1918 | 250 | 0.934838319 | 0.982 | 0.358144298 |
| 39 | 1918 | 500 | 0.977487216 | 0.948 | 0.358144298 |
| 40 | 1918 | 1000 | 0.848694793 | 0.796 | 0.177812899 |
| 41 | 1918 | 2000 | 0.551192399 | 0.644 | 0.220540145 |
| 42 | 1918 | 4000 | 0.907581852 | 0.754 | 0.391082362 |
| 43 | 1899 | AllScales | 0.86326558 | 0.796 | 0.220540145 |
| 44 | 1899 | 250 | 0.900905705 | 0.99 | 0.270185619 |
| 45 | 1899 | 500 | 0.954218084 | 0.988 | 0.244477706 |
| 46 | 1899 | 1000 | 0.960040148 | 0.874 | 0.158918458 |
| 47 | 1899 | 2000 | 0.841057243 | 0.812 | 0.297691291 |
| 48 | 1899 | 4000 | 0.915906812 | 0.944 | 0.177812899 |

Pvalue >0.05 indicates non-significance at 95% interval. The values are based on full versions of the models using AHA values per time step (Spdat~ AHA(Time_step, Scale)) and show no violation of the assumptions of uniformity of residuals, no over or under dispersion and models show expected number of outliers based on the sample size.

**S11. AUC values from a nonlinear (square term) models for species occurrences based on HMD and AHA.**


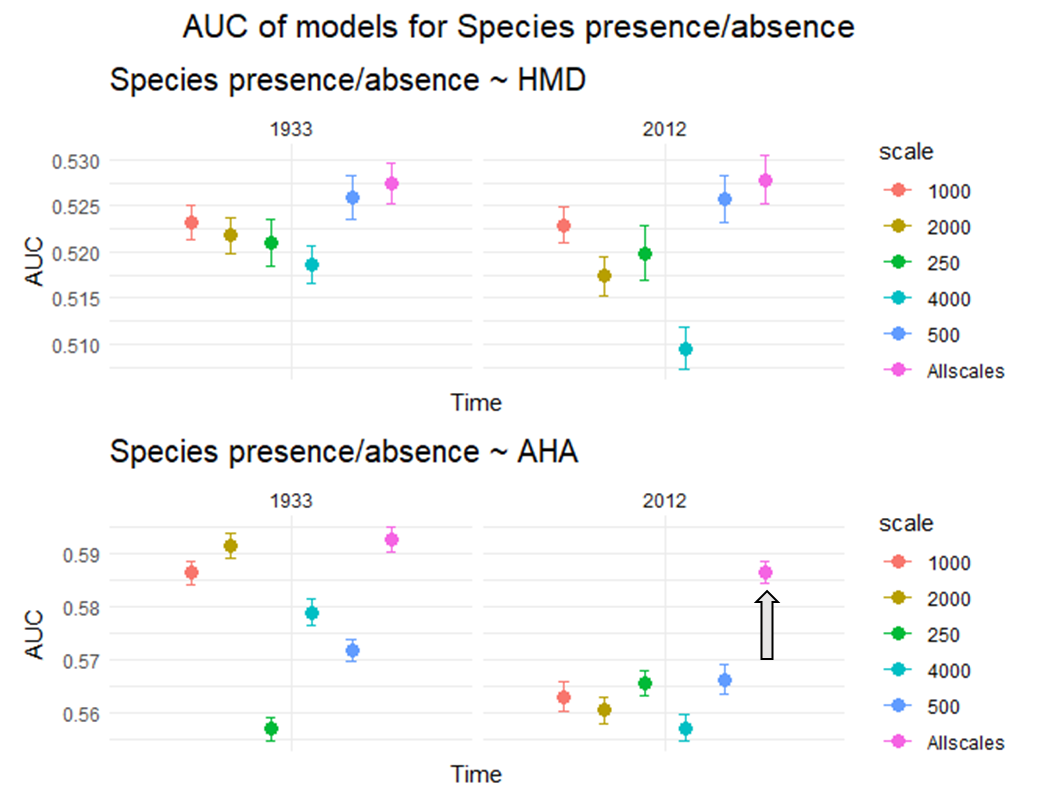


**S12. Table showing GLM model outputs (mean values from 30% subsampling repeated 25 times) for different historical time-steps for the model of structure: Species presence-absence ~ AHA _(per scale per time-step); where_ AHA = Available Habitat Amount.** Effect size is calculated as the logit inverse of the predictor beta coefficient multiplied by the standard deviation.

|  | **Scale** | **Time_step** | **mean_AIC** | **sd_AIC** | **mean_AUC** | **sd_AUC** | **mean_Effsize** | **sd_Effsize** | **mean_coeff** | **sd_coeff** | **mean_Pvalue** |
| --- | --- | --- | --- | --- | --- | --- | --- | --- | --- | --- | --- |
| 1 | 250 | 1899 | 54766 | 18.67 | 0.54 | 0.002 | 0.53 | 0.002 | 0.47 | 0.03 | < 10^-5^ |
| 2 | 250 | 1918 | 54600 | 24.35 | 0.55 | 0.002 | 0.55 | 0.002 | 0.71 | 0.03 | < 10^-5^ |
| 3 | 250 | 1933 | 54549 | 30.79 | 0.56 | 0.002 | 0.55 | 0.002 | 0.80 | 0.03 | < 10^-5^ |
| 4 | 250 | 1959 | 54583 | 28.29 | 0.55 | 0.002 | 0.55 | 0.002 | 0.83 | 0.03 | < 10^-5^ |
| 5 | 250 | 1970 | 54586 | 28.76 | 0.56 | 0.002 | 0.55 | 0.002 | 0.87 | 0.04 | < 10^-5^ |
| 6 | 250 | 1978 | 54521 | 34.99 | 0.56 | 0.002 | 0.55 | 0.002 | 0.95 | 0.04 | < 10^-5^ |
| 7 | 250 | 1992 | 54561 | 29.53 | 0.56 | 0.002 | 0.55 | 0.002 | 0.93 | 0.04 | < 10^-5^ |
| 8 | 250 | 2012 | 54569 | 29.20 | 0.56 | 0.002 | 0.55 | 0.002 | 0.84 | 0.03 | < 10^-5^ |
| 9 | 500 | 1899 | 54655 | 24.79 | 0.55 | 0.002 | 0.54 | 0.002 | 0.83 | 0.04 | < 10^-5^ |
| 10 | 500 | 1918 | 54407 | 33.90 | 0.57 | 0.002 | 0.56 | 0.002 | 1.31 | 0.04 | < 10^-5^ |
| 11 | 500 | 1933 | 54310 | 39.83 | 0.57 | 0.002 | 0.56 | 0.002 | 1.56 | 0.05 | < 10^-5^ |
| 12 | 500 | 1959 | 54309 | 46.36 | 0.57 | 0.002 | 0.56 | 0.002 | 1.80 | 0.07 | < 10^-5^ |
| 13 | 500 | 1970 | 54379 | 38.14 | 0.57 | 0.002 | 0.56 | 0.002 | 1.84 | 0.07 | < 10^-5^ |
| 14 | 500 | 1978 | 54328 | 43.46 | 0.57 | 0.002 | 0.56 | 0.002 | 1.95 | 0.08 | < 10^-5^ |
| 15 | 500 | 1992 | 54446 | 39.06 | 0.57 | 0.003 | 0.56 | 0.002 | 1.75 | 0.08 | < 10^-5^ |
| 16 | 500 | 2012 | 54634 | 30.01 | 0.56 | 0.003 | 0.54 | 0.002 | 1.23 | 0.06 | < 10^-5^ |
| 17 | 1000 | 1899 | 54505 | 31.21 | 0.56 | 0.002 | 0.55 | 0.002 | 1.48 | 0.06 | < 10^-5^ |
| 18 | 1000 | 1918 | 54168 | 39.93 | 0.58 | 0.002 | 0.57 | 0.002 | 2.35 | 0.07 | < 10^-5^ |
| 19 | 1000 | 1933 | 53969 | 44.61 | 0.59 | 0.002 | 0.58 | 0.002 | 2.82 | 0.07 | < 10^-5^ |
| 20 | 1000 | 1959 | 53961 | 55.02 | 0.58 | 0.002 | 0.58 | 0.002 | 2.64 | 0.08 | < 10^-5^ |
| 21 | 1000 | 1970 | 54081 | 46.56 | 0.58 | 0.002 | 0.57 | 0.002 | 2.53 | 0.08 | < 10^-5^ |
| 22 | 1000 | 1978 | 54081 | 55.33 | 0.58 | 0.003 | 0.57 | 0.003 | 2.55 | 0.09 | < 10^-5^ |
| 23 | 1000 | 1992 | 54257 | 54.43 | 0.57 | 0.003 | 0.57 | 0.003 | 2.02 | 0.09 | < 10^-5^ |
| 24 | 1000 | 2012 | 54564 | 36.36 | 0.56 | 0.003 | 0.55 | 0.002 | 1.42 | 0.07 | < 10^-5^ |
| 25 | 2000 | 1899 | 54471 | 36.17 | 0.56 | 0.002 | 0.56 | 0.002 | 2.13 | 0.09 | < 10^-5^ |
| 26 | 2000 | 1918 | 54009 | 49.94 | 0.59 | 0.002 | 0.58 | 0.002 | 3.59 | 0.11 | < 10^-5^ |
| 27 | 2000 | 1933 | 53760 | 54.94 | 0.59 | 0.002 | 0.59 | 0.002 | 3.69 | 0.09 | < 10^-5^ |
| 28 | 2000 | 1959 | 53884 | 66.12 | 0.58 | 0.003 | 0.58 | 0.003 | 3.30 | 0.11 | < 10^-5^ |
| 29 | 2000 | 1970 | 53988 | 53.19 | 0.58 | 0.003 | 0.58 | 0.002 | 2.88 | 0.08 | < 10^-5^ |
| 30 | 2000 | 1978 | 54021 | 57.03 | 0.58 | 0.003 | 0.58 | 0.002 | 2.56 | 0.08 | < 10^-5^ |
| 31 | 2000 | 1992 | 54241 | 55.06 | 0.57 | 0.003 | 0.57 | 0.003 | 2.02 | 0.08 | < 10^-5^ |
| 32 | 2000 | 2012 | 54585 | 34.45 | 0.55 | 0.003 | 0.55 | 0.002 | 1.51 | 0.07 | < 10^-5^ |
| 33 | 4000 | 1899 | 54649 | 28.24 | 0.54 | 0.002 | 0.54 | 0.002 | 1.77 | 0.09 | < 10^-5^ |
| 34 | 4000 | 1918 | 54212 | 46.28 | 0.57 | 0.002 | 0.57 | 0.002 | 3.31 | 0.11 | < 10^-5^ |
| 35 | 4000 | 1933 | 53990 | 50.60 | 0.58 | 0.002 | 0.58 | 0.002 | 3.36 | 0.10 | < 10^-5^ |
| 36 | 4000 | 1959 | 54142 | 52.43 | 0.57 | 0.003 | 0.57 | 0.002 | 2.69 | 0.09 | < 10^-5^ |
| 37 | 4000 | 1970 | 54233 | 45.30 | 0.56 | 0.002 | 0.57 | 0.002 | 2.39 | 0.08 | < 10^-5^ |
| 38 | 4000 | 1978 | 54293 | 45.59 | 0.56 | 0.003 | 0.56 | 0.002 | 2.08 | 0.07 | < 10^-5^ |
| 39 | 4000 | 1992 | 54482 | 40.76 | 0.55 | 0.003 | 0.55 | 0.002 | 1.64 | 0.07 | < 10^-5^ |
| 40 | 4000 | 2012 | 54755 | 24.47 | 0.53 | 0.003 | 0.53 | 0.002 | 0.99 | 0.07 | < 10^-5^ |
| 41 | AllScales | 1899 | 54433 | 36.07 | 0.57 | 0.002 | 0.55 | 0.002 | 0.00 | 0.05 | 0.065 |
| 42 | AllScales | 1918 | 54004 | 48.97 | 0.59 | 0.002 | 0.58 | 0.002 | 0.02 | 0.06 | 0.067 |
| 43 | AllScales | 1933 | 53749 | 53.49 | 0.59 | 0.002 | 0.59 | 0.003 | -0.02 | 0.07 | 0.069 |
| 44 | AllScales | 1959 | 53835 | 66.61 | 0.59 | 0.003 | 0.59 | 0.003 | -0.16 | 0.06 | 0.074 |
| 45 | AllScales | 1970 | 53956 | 52.55 | 0.58 | 0.002 | 0.58 | 0.003 | -0.04 | 0.06 | 0.078 |
| 46 | AllScales | 1978 | 53968 | 59.28 | 0.58 | 0.003 | 0.58 | 0.003 | 0.06 | 0.06 | 0.078 |
| 47 | AllScales | 1992 | 54168 | 57.12 | 0.57 | 0.003 | 0.57 | 0.003 | 0.18 | 0.07 | 0.081 |
| 48 | AllScales | 2012 | 54415 | 38.28 | 0.57 | 0.002 | 0.56 | 0.003 | 0.61 | 0.04 | 0.074 |

**S13. Table showing GLM model outputs (mean values from 30% subsampling repeated 25 times) for different historical time-steps for the model of structure: Species presence-absence ~ HMD _(per scale per time-step); where HMD =_ Human Modification Density.** Effect size is calculated as the logit inverse of the predictor beta coefficient multiplied by the standard deviation.

|  | **Scale** | **Time_step** | **mean_AIC** | **sd_AIC** | **mean_AUC** | **sd_AUC** | **mean_Effsize** | **sd_Effsize** | **mean_coeff** | **sd_coeff** | **mean_Pvalue** |
| --- | --- | --- | --- | --- | --- | --- | --- | --- | --- | --- | --- |
| 1 | 250 | 1899 | 54906 | 11 | 0.52 | 0.003 | 0.48 | 0.002 | -0.66 | 0.10 | < 10^-5^ |
| 2 | 250 | 1918 | 54883 | 12 | 0.52 | 0.002 | 0.48 | 0.002 | -0.82 | 0.08 | < 10^-5^ |
| 3 | 250 | 1933 | 54876 | 13 | 0.52 | 0.003 | 0.48 | 0.002 | -0.85 | 0.08 | < 10^-5^ |
| 4 | 250 | 1959 | 54864 | 14 | 0.52 | 0.003 | 0.48 | 0.002 | -0.93 | 0.09 | < 10^-5^ |
| 5 | 250 | 1970 | 54868 | 14 | 0.52 | 0.003 | 0.48 | 0.002 | -0.81 | 0.07 | < 10^-5^ |
| 6 | 250 | 1978 | 54863 | 14 | 0.52 | 0.003 | 0.48 | 0.002 | -0.81 | 0.07 | < 10^-5^ |
| 7 | 250 | 1992 | 54875 | 13 | 0.52 | 0.003 | 0.48 | 0.002 | -0.69 | 0.07 | < 10^-5^ |
| 8 | 250 | 2012 | 54871 | 13 | 0.52 | 0.003 | 0.48 | 0.002 | -0.51 | 0.05 | < 10^-5^ |
| 9 | 500 | 1899 | 54884 | 11 | 0.52 | 0.002 | 0.48 | 0.002 | -0.95 | 0.09 | < 10^-5^ |
| 10 | 500 | 1918 | 54854 | 12 | 0.52 | 0.002 | 0.48 | 0.002 | -1.20 | 0.08 | < 10^-5^ |
| 11 | 500 | 1933 | 54845 | 16 | 0.53 | 0.002 | 0.48 | 0.002 | -1.23 | 0.10 | < 10^-5^ |
| 12 | 500 | 1959 | 54831 | 17 | 0.53 | 0.002 | 0.47 | 0.002 | -1.29 | 0.10 | < 10^-5^ |
| 13 | 500 | 1970 | 54842 | 16 | 0.53 | 0.002 | 0.47 | 0.002 | -1.09 | 0.09 | < 10^-5^ |
| 14 | 500 | 1978 | 54834 | 17 | 0.53 | 0.003 | 0.47 | 0.002 | -1.06 | 0.08 | < 10^-5^ |
| 15 | 500 | 1992 | 54848 | 16 | 0.53 | 0.002 | 0.48 | 0.002 | -0.92 | 0.07 | < 10^-5^ |
| 16 | 500 | 2012 | 54845 | 16 | 0.53 | 0.003 | 0.48 | 0.002 | -0.69 | 0.06 | < 10^-5^ |
| 17 | 1000 | 1899 | 54890 | 9 | 0.51 | 0.002 | 0.48 | 0.002 | -1.07 | 0.09 | < 10^-5^ |
| 18 | 1000 | 1918 | 54859 | 13 | 0.52 | 0.002 | 0.48 | 0.002 | -1.35 | 0.10 | < 10^-5^ |
| 19 | 1000 | 1933 | 54850 | 14 | 0.52 | 0.002 | 0.48 | 0.002 | -1.39 | 0.10 | < 10^-5^ |
| 20 | 1000 | 1959 | 54841 | 16 | 0.53 | 0.002 | 0.47 | 0.002 | -1.37 | 0.10 | < 10^-5^ |
| 21 | 1000 | 1970 | 54858 | 14 | 0.52 | 0.002 | 0.48 | 0.002 | -1.08 | 0.09 | < 10^-5^ |
| 22 | 1000 | 1978 | 54849 | 15 | 0.53 | 0.002 | 0.48 | 0.002 | -1.06 | 0.08 | < 10^-5^ |
| 23 | 1000 | 1992 | 54865 | 13 | 0.52 | 0.002 | 0.48 | 0.002 | -0.89 | 0.07 | < 10^-5^ |
| 24 | 1000 | 2012 | 54867 | 13 | 0.52 | 0.002 | 0.48 | 0.002 | -0.67 | 0.06 | < 10^-5^ |
| 25 | 2000 | 1899 | 54901 | 7 | 0.51 | 0.004 | 0.48 | 0.001 | -1.03 | 0.09 | < 10^-5^ |
| 26 | 2000 | 1918 | 54859 | 13 | 0.52 | 0.002 | 0.48 | 0.002 | -1.58 | 0.11 | < 10^-5^ |
| 27 | 2000 | 1933 | 54850 | 14 | 0.52 | 0.002 | 0.48 | 0.002 | -1.54 | 0.11 | < 10^-5^ |
| 28 | 2000 | 1959 | 54842 | 16 | 0.53 | 0.002 | 0.47 | 0.002 | -1.50 | 0.11 | < 10^-5^ |
| 29 | 2000 | 1970 | 54867 | 14 | 0.52 | 0.002 | 0.48 | 0.002 | -1.12 | 0.10 | < 10^-5^ |
| 30 | 2000 | 1978 | 54863 | 14 | 0.53 | 0.002 | 0.48 | 0.002 | -1.05 | 0.09 | < 10^-5^ |
| 31 | 2000 | 1992 | 54884 | 13 | 0.52 | 0.002 | 0.48 | 0.002 | -0.85 | 0.09 | < 10^-5^ |
| 32 | 2000 | 2012 | 54894 | 12 | 0.52 | 0.002 | 0.48 | 0.002 | -0.59 | 0.07 | < 10^-5^ |
| 33 | 4000 | 1899 | 54935 | 4 | 0.51 | 0.002 | 0.49 | 0.002 | -0.34 | 0.09 | < 10^-5^ |
| 34 | 4000 | 1918 | 54903 | 9 | 0.52 | 0.002 | 0.48 | 0.002 | -1.01 | 0.11 | < 10^-5^ |
| 35 | 4000 | 1933 | 54895 | 9 | 0.52 | 0.002 | 0.48 | 0.002 | -1.09 | 0.11 | < 10^-5^ |
| 36 | 4000 | 1959 | 54880 | 11 | 0.53 | 0.002 | 0.48 | 0.002 | -1.11 | 0.09 | < 10^-5^ |
| 37 | 4000 | 1970 | 54904 | 8 | 0.52 | 0.002 | 0.48 | 0.002 | -0.78 | 0.08 | < 10^-5^ |
| 38 | 4000 | 1978 | 54903 | 9 | 0.52 | 0.002 | 0.48 | 0.002 | -0.73 | 0.08 | < 10^-5^ |
| 39 | 4000 | 1992 | 54922 | 6 | 0.51 | 0.002 | 0.49 | 0.002 | -0.50 | 0.08 | < 10^-5^ |
| 40 | 4000 | 2012 | 54934 | 4 | 0.51 | 0.002 | 0.49 | 0.002 | -0.25 | 0.07 | 0.019 |
| 41 | AllScales | 1899 | 54871 | 12 | 0.52 | 0.003 | 0.48 | 0.002 | -0.15 | 0.14 | 0.166 |
| 42 | AllScales | 1918 | 54830 | 15 | 0.52 | 0.002 | 0.47 | 0.002 | -0.21 | 0.13 | 0.165 |
| 43 | AllScales | 1933 | 54820 | 17 | 0.53 | 0.002 | 0.47 | 0.002 | -0.20 | 0.12 | 0.164 |
| 44 | AllScales | 1959 | 54808 | 19 | 0.53 | 0.002 | 0.46 | 0.002 | -0.18 | 0.13 | 0.176 |
| 45 | AllScales | 1970 | 54833 | 18 | 0.53 | 0.002 | 0.47 | 0.002 | -0.16 | 0.11 | 0.165 |
| 46 | AllScales | 1978 | 54825 | 19 | 0.53 | 0.002 | 0.47 | 0.002 | -0.15 | 0.11 | 0.160 |
| 47 | AllScales | 1992 | 54843 | 18 | 0.53 | 0.002 | 0.47 | 0.002 | -0.08 | 0.11 | 0.153 |
| 48 | AllScales | 2012 | 54835 | 18.69 | 0.53 | 0.003 | 0.48 | 0.002 | -0.08 | 0.08 | 0.109 |

**S14: Table showing GLM model outputs (Full models with no subsampling) for different historical time-steps for the model of structure: Species presence-absence ~ AHA _(per scale per time-step); where_ AHA = Available Habitat Amount**

|  | **Time_step** | **scale** | **coeff** | **AIC** | **AUC** | **Eff_size** | **pval** |
| --- | --- | --- | --- | --- | --- | --- | --- |
| 1 | 2012 | 250 | 0.79 | 133979 | 0.56 | 0.55 | < 10^-5^ |
| 2 | 2012 | 500 | 1.13 | 134127 | 0.56 | 0.54 | < 10^-5^ |
| 3 | 2012 | 1000 | 1.30 | 133984 | 0.56 | 0.55 | < 10^-5^ |
| 4 | 2012 | 2000 | 1.37 | 134037 | 0.55 | 0.55 | < 10^-5^ |
| 5 | 2012 | 4000 | 0.90 | 134377 | 0.53 | 0.53 | < 10^-5^ |
| 6 | 1992 | 250 | 0.90 | 133938 | 0.56 | 0.55 | < 10^-5^ |
| 7 | 1992 | 500 | 1.66 | 133708 | 0.57 | 0.55 | < 10^-5^ |
| 8 | 1992 | 1000 | 1.91 | 133314 | 0.57 | 0.56 | < 10^-5^ |
| 9 | 1992 | 2000 | 1.90 | 133303 | 0.57 | 0.56 | < 10^-5^ |
| 10 | 1992 | 4000 | 1.52 | 133831 | 0.55 | 0.55 | < 10^-5^ |
| 11 | 1978 | 250 | 0.93 | 133850 | 0.56 | 0.55 | < 10^-5^ |
| 12 | 1978 | 500 | 1.87 | 133447 | 0.57 | 0.56 | < 10^-5^ |
| 13 | 1978 | 1000 | 2.44 | 132920 | 0.58 | 0.57 | < 10^-5^ |
| 14 | 1978 | 2000 | 2.44 | 132818 | 0.58 | 0.57 | < 10^-5^ |
| 15 | 1978 | 4000 | 1.97 | 133425 | 0.56 | 0.56 | < 10^-5^ |
| 16 | 1970 | 250 | 0.85 | 133987 | 0.55 | 0.55 | < 10^-5^ |
| 17 | 1970 | 500 | 1.78 | 133539 | 0.57 | 0.56 | < 10^-5^ |
| 18 | 1970 | 1000 | 2.44 | 132905 | 0.58 | 0.57 | < 10^-5^ |
| 19 | 1970 | 2000 | 2.78 | 132736 | 0.58 | 0.58 | < 10^-5^ |
| 20 | 1970 | 4000 | 2.29 | 133291 | 0.56 | 0.57 | < 10^-5^ |
| 21 | 1959 | 250 | 0.82 | 133978 | 0.55 | 0.55 | < 10^-5^ |
| 22 | 1959 | 500 | 1.77 | 133367 | 0.57 | 0.56 | < 10^-5^ |
| 23 | 1959 | 1000 | 2.57 | 132619 | 0.58 | 0.58 | < 10^-5^ |
| 24 | 1959 | 2000 | 3.21 | 132480 | 0.58 | 0.58 | < 10^-5^ |
| 25 | 1959 | 4000 | 2.61 | 133071 | 0.57 | 0.57 | < 10^-5^ |
| 26 | 1933 | 250 | 0.79 | 133908 | 0.56 | 0.55 | < 10^-5^ |
| 27 | 1933 | 500 | 1.52 | 133382 | 0.57 | 0.56 | < 10^-5^ |
| 28 | 1933 | 1000 | 2.72 | 132650 | 0.59 | 0.57 | < 10^-5^ |
| 29 | 1933 | 2000 | 3.53 | 132223 | 0.59 | 0.58 | < 10^-5^ |
| 30 | 1933 | 4000 | 3.24 | 132732 | 0.58 | 0.58 | < 10^-5^ |
| 31 | 1918 | 250 | 0.71 | 134006 | 0.55 | 0.55 | < 10^-5^ |
| 32 | 1918 | 500 | 1.29 | 133585 | 0.57 | 0.56 | < 10^-5^ |
| 33 | 1918 | 1000 | 2.24 | 133099 | 0.58 | 0.57 | < 10^-5^ |
| 34 | 1918 | 2000 | 3.31 | 132809 | 0.58 | 0.57 | < 10^-5^ |
| 35 | 1918 | 4000 | 3.07 | 133245 | 0.57 | 0.56 | < 10^-5^ |
| 36 | 1899 | 250 | 0.46 | 134389 | 0.54 | 0.53 | < 10^-5^ |
| 37 | 1899 | 500 | 0.80 | 134156 | 0.55 | 0.54 | < 10^-5^ |
| 38 | 1899 | 1000 | 1.38 | 133856 | 0.56 | 0.55 | < 10^-5^ |
| 39 | 1899 | 2000 | 1.92 | 133808 | 0.56 | 0.55 | < 10^-5^ |
| 40 | 1899 | 4000 | 1.65 | 134157 | 0.54 | 0.54 | < 10^-5^ |
| 41 | 2012 | AllScales | NA | 133656 | 0.57 | 0.55 | 0.05 |
| 42 | 1992 | AllScales | NA | 133113 | 0.58 | 0.57 | 0.05 |
| 43 | 1978 | AllScales | NA | 132679 | 0.58 | 0.58 | 0.05 |
| 44 | 1970 | AllScales | NA | 132647 | 0.58 | 0.58 | 0.05 |
| 45 | 1959 | AllScales | NA | 132355 | 0.59 | 0.58 | 0.05 |
| 46 | 1933 | AllScales | NA | 132199 | 0.59 | 0.59 | 0.05 |
| 47 | 1918 | AllScales | NA | 132786 | 0.59 | 0.57 | 0.05 |
| 48 | 1899 | AllScales | NA | 133725 | 0.57 | 0.55 | 0.04 |

**S15: Software workflow, packages and functions used in analysis**

**
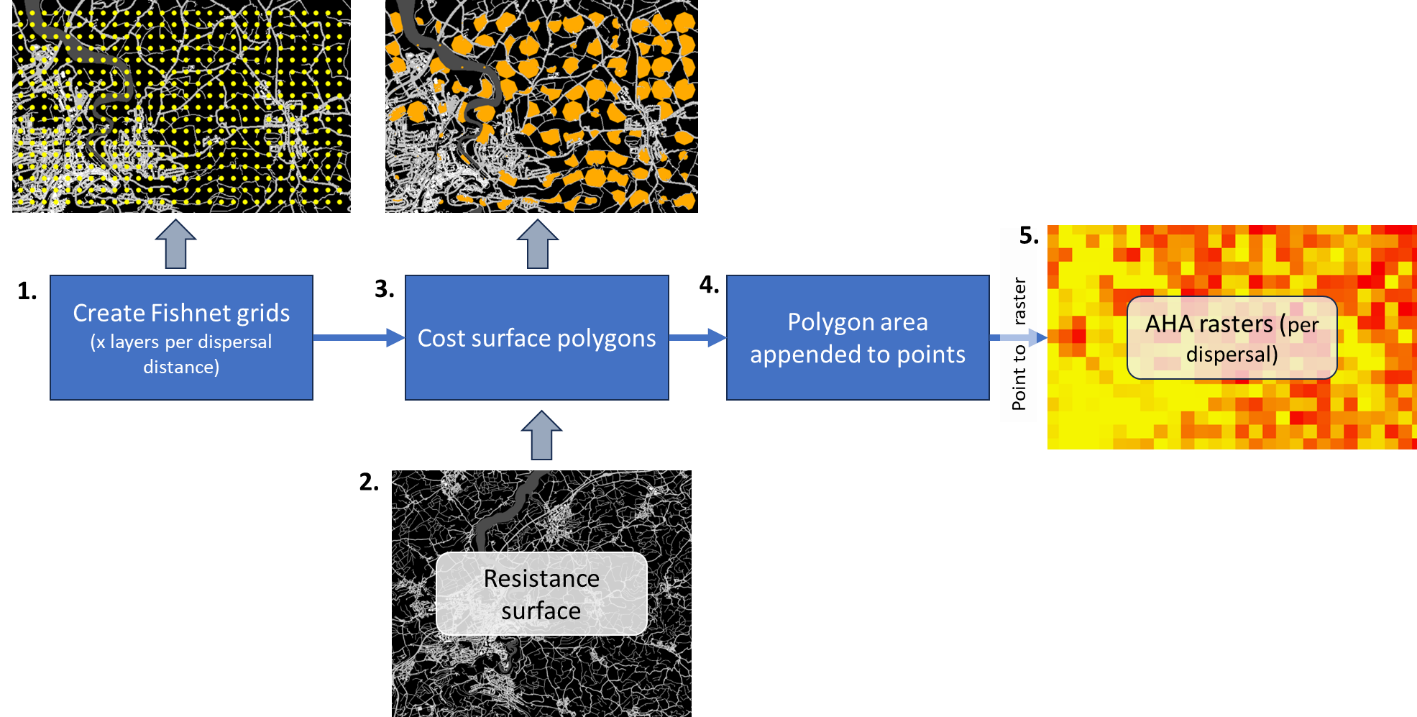
**

| **Process** | **Description** | **Major Functions used** | **Code ref** |
| --- | --- | --- | --- |
| **Calculating amount of habitat available (AHA)** | |  |  |
| 1. Create fishnet for multiple scales | Since we use "CostDistance" function from arcpy that finds the distance between multiple points in the same shapefile, we leveraged this fast function by creating points in a grid-like manner across the Swiss plateau. These grids are spaced based on 2 * dispersal ability thresholds of 250,500,1000,2000 and 4000 so as to gain a radius of each dispersal ability distance. However, since we want the final metric to be computed for every 250 by 250 resolution, we shift each grid of the dispersal thresholds by 250 in all directions to gain multiple shapefiles that together span the entire study area. This implies that there are 4 grid layers with spacing of 500 (i.e. 2 *250) to get the 500 m dispersal from each 250 by 250 pixel centre. | arcpy.CreateFishnet_management() in python | *Create_fishnet.py* |
| 2. Resistance surface creation | We combine the roads (buffered to chosen widths; ref section 2.2) and the buildings from the source data (Example shows Swiss TLM) and reclassified the landcover to roads =25 and buildings =50 | terra::classify() in R | *__* |
| 3. Cost surface creation | Then we run the CostDistance function for each set of grids with the corresponding maxDistance of 250,500,1000, 2000 and 4000m. These cost distance surfaces are converted to polygons truncated at the maximum dispersal and saved to save space. | arcpy.CostDistance() in python | *Cost_surface_creation.py* |
| 4.Cost surface area estimation | The created polygons are then split and then their area attributes added as a field. | arcpy spatial analysis and management tools in python | *Cost_polygon_area.py* |
| 5. Create final AHA layers | These areas are then attributed back to the point layers and then all the point layers are combined to form the final AHA layer per dispersal scale | arcpy spatial analysis and management tools in python | *Create_final_AHA.py* |
| **Clustering and statistical analysis** | |  |  |
| Clustering | We perform a multidimensional Kmeans clustering on the time-series data across the 5 dispersals and 8 time steps. We also ran a spatial Kmeans clustering on the contemporary data, and mapped its labels to the time series clustering based on ____algorithm. | tslearn.clustering.TimeSerieskmeans() kneed::KneeLocator() sklearn::Kmeans() scipy.optimize.linear_sum_assignment() in python | __ |
| Cluster stats | We assign a cluster class to each landscape quality hectare and plot barplots of the frequency of high quality pixels per cluster class. We also define a function to test the p-value based on a randomisation based permutation test of the cluster labesls | base R functions and Ggplot in R | __ |
| GLM | We ran Generalised linear models using binomial reponse on species presence absence data | lme4::glm() ; proc::roc(),auc() in R | __ |
| HMD creation | We calculated the Human modificatoin density as the density of road and building pixels in square grid sizes equalent to dispersal ability in non resistance landscape. | pytorch()::AvgPool2d in python |  |

**S16: Table showing the time taken for the AHA metric creation for different spatial scales of input, run on a workstation with intel®Core i710700 CPU with 32 GB RAM.**

| **Process** | **Spatial Scale** | **Time taken (minutes)** |  |
| --- | --- | --- | --- |
| 1. Creating fishnets | ~40 sq km | 7 |  |
|  | Swiss plateau (9109 sq km) | 13 |  |
|  |  |  |  |
| 2.Cost surface creation | ~40 sq km* | 4 |  |
|  | Swiss plateau (9109 sq km) | ~900 |  |
|  |  |  |  |
| 3. Cost surface Area estimation | ~40 sq km* | 1.8 |  |
|  | Swiss plateau (9109 sq km) | ~210 |  |
|  |  |  |  |
| 4. Create final AHA layers | ~40 sq km* | <1 |  |
|  | Swiss plateau (9109 sq km) | ~10 |  |
| * only computes 4 dispersal scales since 4000m is beyond study extents | | |  |
|  |  |  |  |
